# What Hides in the Heights? The Case of the Iberian Endemism *Bromus picoeuropeanus*

**DOI:** 10.3390/plants12071531

**Published:** 2023-04-01

**Authors:** Claudia González-Toral, Herminio S. Nava, José Antonio Fernández Prieto, Eduardo Cires

**Affiliations:** 1 Department of Organisms and Systems Biology, University of Oviedo, C/Catedrático Rodrigo Uría s/n, 33071 Oviedo, Spain; 2 Institute of Natural Resources and Territorial Planning (INDUROT), Campus de Mieres, C/Gonzalo Gutiérrez Quirós s/n, 33600 Mieres, Spain

**Keywords:** *Bromus*, Cantabrian Mountains, external transcribed spacer (ETS), internal transcribed spacer (ITS), Poaceae, trnL

## Abstract

*Bromus picoeuropeanus* is a recently described species belonging to a complex genus of grasses. It inhabits stony soils at heights ranging from 1600 to 2200 m in Picos de Europa (Cantabrian Mountains, northern Spain). This species is morphologically very similar to *B. erectus*, partially sharing its presumed distribution range. We aim to determine the relationship between these species and their altitudinal ranges in Picos de Europa and the Cantabrian Mountains by conducting phylogenetic analyses based on nuclear (ETS and ITS) and chloroplastic (trnL) markers. Phylogenetic trees were inferred by Maximum Likelihood and Bayesian Inference. Haplotype networks were estimated based on the plastid marker. Although the ITS topologies could not generate exclusive clades for these species, the ETS analyses generated highly supported *B. picoeuropeanus* exclusive clades, which included locations outside its altitudinal putative range. The ETS-ITS and ETS-ITS-trnL topologies generated *B. picoeuropeanus* exclusive clades, whereas the trnL-based trees and haplotype networks were unable to discriminate *B. erectus* and *B. picoeuropeanus*. This evidence suggests that *B. picoeuropeanus* is a separate species with a larger distribution than previously thought, opening new questions regarding the evolution of *B. erectus* and other similar species in European mountainous systems. However, more information is needed regarding *B. picoeuropeanus* susceptibility to temperature rises.

## 1. Introduction

Climate change as a consequence of anthropogenic activities is one of the processes driving the current Biological Diversity Crisis [1,2,3,4]). This has resulted, among other things, in high plant extinction rates [5], these extinctions being more significant in hotspots of biodiversity [6]. In this context, mountainous regions, one of the habitats comprising the most plant endemism, especially in Europe [7], have been reported to experience a faster warming process than other habitats [8,9]. These temperature rises may cause shifts in the range of distribution of mountainous species [10] which, in the case of the endemism of high mountain species, represent a double danger as they face not only a reduction in their potential range of distribution, but also new competitors from lower lands [11,12].

*Bromus* L. (1753) is a large genus of annual, biannual, and perennial grasses belonging to the Poaceae family, which is estimated to comprise around 140–200 species distributed throughout both hemispheres [13,14,15,16,17,18] and contains several high mountains species such as *Bromus carinatus* Hook. and Arn. (1840) [17,19,20,21]. This heterogeneous and reticulated group has frequent hybridization and polyploidization events and high morphological plasticity [17,21], which accounts for the different taxonomic treatments of *Bromus* divided into several sections [22] or subgenera [23], or even splitted into different genera [24,25]. Consequently, numerous studies based on morphological [13,20,26], cytological [27,28,29] and molecular data [30,31,32] have been conducted.

In the Iberian Peninsula, Acedo and Llamas [13,29] described 26 different *Bromus* species, 18 of which have been reported to occur in the mountainous systems of the north of Spain [33,34,35]. Among those 18, the Eurasian perennial tetraploid grass *Bromus erectus* Huds. (1762) *sensu lato* (s. l.) is a highly variable group of microspecies that inhabits mountainous areas of central Europe, the Atlantic and Mediterranean European Basins, including the British Islands as well as in Iran and Tibet [17,36,37,38,39,40,41]. *B. erectus* s. l. has been reported to have evolved in at least two glacial refugia of Central Europe [40,42], something that could be related to the occurrence of many microspecies in its mountainous regions. For instance, *B. erectus sensu stricto* (s. s.) has been described by Bačič and Jogan [38] to inhabit lower lands up to an altitude of 600 m a. s. l. to experience an altitudinal segregation in some regions of the Slovenian Alps with another two closely related species of the “*B. erectus* group”, i.e., *B. transylvanicus* Steud. (1854) and *Bromus condensatus* Hack. (1879), previously considered a subspecies or synonyms of *B. erectus* (i.e., *B. erectus* s. l.) [41]. Nevertheless, although *B. erectus* s. l. has been subject to morphological [43,44], cytological [45] and molecular studies [40,46], its microspecies remain relatively unknown [38].

**Figure 1:**
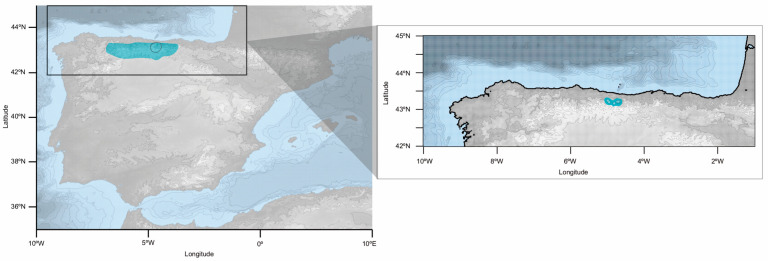
*Bromus picoeuropeanus* range in Picos de Europa, encircled in black, within the Cantabrian Mountains, highlighted in blue. In the box, the blue dots represent the localities of *B. picoeuropeanus* reported by Acedo and Llamas [29]. The base map was obtained using marmap R Pakage [47] and edited using Inkscape [48].

In this context of altitudinal segregation of members of the *B. erectus* complex, *B. picoeuropeanus* Acedo and Llamas (2019) has been recently described in the mountainous regions of the north of Spain [29] (see Figure 1). Morphologically, *B. picoeuropeanus* can be distinguished from *B. erectus* by the presence of well-developed rhizomes, its “loosely tufted” habit, its shorter height (no more than 40 cm) and its truncated or rounded ligule, among other features [29]. *Bromus picoeuropeanus* is endemic to the mountainous region of Picos de Europa in the Cantabrian Mountains (north Spain) and inhabits stony soils from 1600 to 2200 m a. s. l. [29]. On the other hand, *B. erectus* s. s. has been reported for the southern Cantabrian Mountains (in León and Palencia) at altitudes ranging from 1490 to 1520 m a. s. l. [13].

**Figure 2:**
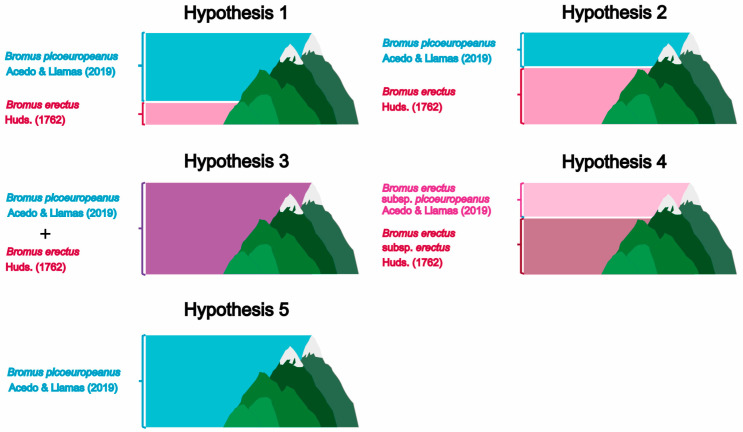
The five hypotheses of the occurrence of *Bromus erectus* s. s. and *Bromus picoeuropeanus* in Picos de Europa. Hypothesis 1 assumes that *B. erectus* s. s. and *B. picoeuropeanus* are two distinct species, being the altitudinal range of *B. erectus* s. s. described by Bačič and Jogan [38]; Hypothesis 2 also assumes the existence of two different species with the altitudinal range given by Acedo and Llamas [29] and Hudson [50]; Hypothesis 3 assumes the existence of two distinct species that would co-occur in Picos de Europa; Hypothesis 4 assumes that *B. picoeuropeanus* would be a subspecies of *B. erectus* s. s. with the altitudinal range proposed by Acedo and Llamas [29] for *B. picoeuropeanus*; Hypothesis 5 assumes the existence of both species, *B. picoeuropeanus* being the only one found in Picos de Europa.

Taking into account the evolutionary history of *B. erectus* s. l. and the fact that the Cantabrian Mountains have been reported to be a glacial refugium for other plant groups [49], the current evidence suggests that a similar segregation to that described by Bačič and Jogan (2001) [38] in the Alps could be found in Picos de Europa regarding *B. erectus* s. s. and *B. picoeuropeanus*. Nevertheless, since no phylogenetic study establishing the relationship has been conducted, we cannot rule out the possibility that *B. picoeuropeanus*, considered within the *B. erectus* complex by Acedo and Llamas [29], is an expression of the plasticity of *B. erectus* s. s. Therefore, we propose five hypotheses to test: (1) *B. erectus* s. s. and *B. picoeuropeanus* are two different species and *B. erectus* s. s. is found at up to 600 m of altitude, (2) *B. erectus* s. s. and *B. picoeuropeanus* are two different species and *B. erectus* s. s. is found at up to approximately 1600 m of altitude, (3) *B. erectus* s. s. and *B. picoeuropeanus* are two different species but there is no altitudinal segregation, (4) *B. picoeuropeanus* is another subspecies *B. erectus* s. s. that can be found at up to 2200 m of altitude and (5) *B. picoeuropeanus* is a different species from *B. erectus* s. s. and is the only one that inhabits the Picos de Europa (see Figure 2). In order to investigate the relationships between the Spanish populations of *B. erectus* s. s. and *B. picoeuropeanus* and their eventual different altitudinal distribution, we carried out molecular analyses based on both nuclear and plastidial markers.

## 2. Results

The main features of the obtained alignments (displayed in Table 1) showed similar numbers of parsimonious-informative sites for ETS and ITS markers, the former having the higher number. On the other hand, the trnL plastid marker showed a lower number of parsimonious-informative sites.

The new ETS sequences of *B. picoeuropeanus* and *B. erectus* from individuals collected in the Cantabrian Mountains and the lowlands of Asturias (Br1-12) presented an indel of 119–121 bp ranging for position from 324 to 451 of the obtained alignment, identical in some cases or almost identical in others to the one presented by several *B. erectus* sequences, including the one collected in Picos de Europa (KJ632441). This insertion was also shared with other species such as *B. sterilis* L. (1753), *B. riparius* Rehmann (1872) or *B. diandrus* Roth (1787). Since the presence of this large indel in various species could influence the position of the *Bromus* samples and their inferred relationships, we performed an additional phylogenetic analysis on the ETS dataset excluding the indel region to determine the influence of this indel on the topology and the branch support (Figure S1).

**Figure 3:**
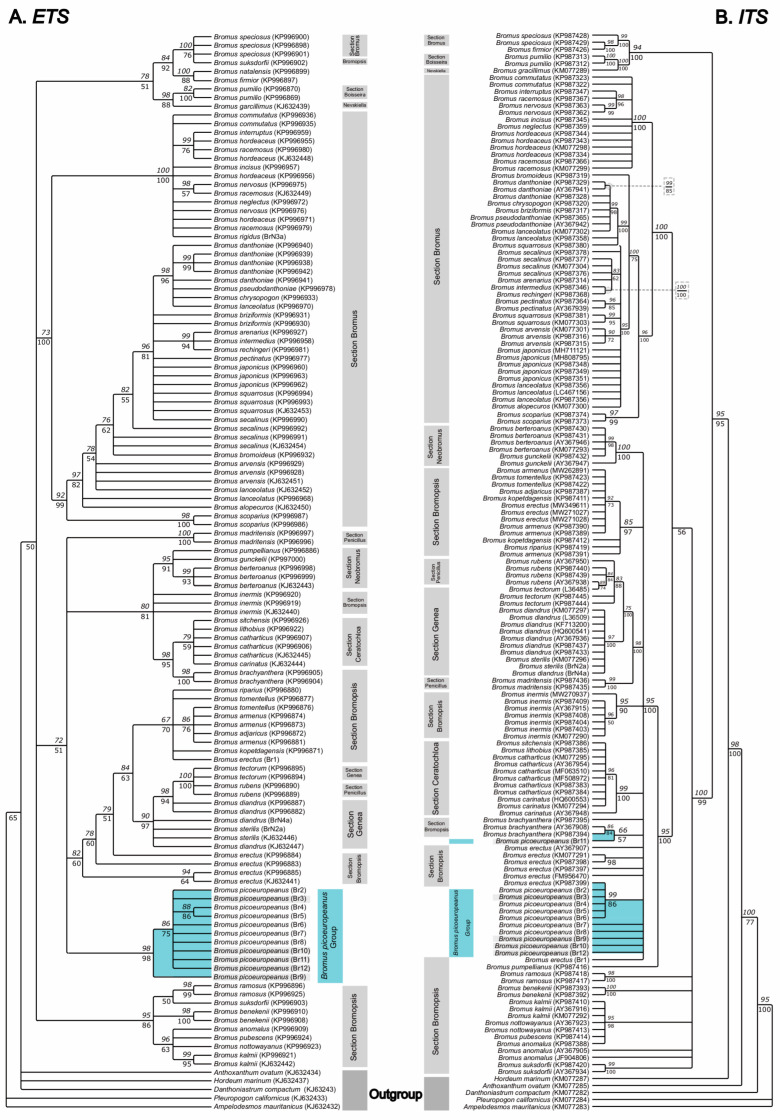
Consensus phylogenetic tree was obtained from ML and BI analyses based on the nuclear marker ETS (**A**) and ITS (**B**). The numbers over the branches correspond to the bootstrap (BS) values from the ML analysis, whereas the numbers under the branches represent the posterior probabilities (PP) obtained during the BI analysis. Br: new *Bromus* samples were generated in this study. The Br samples from the Cantabrian Mountains and from Picos de Europa have been highlighted in blue. The Br sample from lower lands has been highlighted in gray.

The phylogenetic analyses of the ETS dataset (see Figure 3A) identified a Cantabrian Mountains clade formed by the samples identified as *B. picoeuropeanus* (Br2-12) (98 BS-ML, 98 PP-BI), which was independent from the clade containing the *B. erectus* sequences and those of other species which presented a similar large indel (82 BS-ML, 62 PP-BI). The *B. erectus* sequence (Br1) belonged to a moderately supported clade (67 BS-ML, 70 PP-BI), sister (84 BS-ML, 63 PP-BI) to that formed by sequences of *B. erectus*, *B. sterilis*, *B. diandrus*, *B. rubens* L. and *B. tectorum* L. and other species of sections *Genea* and *Penicillius* (90 BS-ML, 97 PP-BI). Both clades, the one containing the *B. picoeuropeanus* sequences (*B. picoeuropeanus* group) and the one containing the *B. erectus* sequences, had a moderately supported sister relationship (72 BS-ML, 51 PP-BI) among them, as well as with the *B. madritensis* L. clade (100 BS-ML, 100 PP-BI) and another large clade containing species of sections *Ceratochloa*, *Bromopsis*, *Penicillius* and *Neobromus*.

The ITS consensus gene tree (see Figure 3B) presented a topology in which neither *B. picoeuropeanus* samples nor *B. erectus* sequences formed an exclusive clade. The *B. picoeuropeanus* and *B. erectus* sequences formed part of a major clade (95 BS-ML, 100 PP-BI) which comprises several subclades of *Bromus* species belonging to sections *Genea*, *Bromopsis*, *Neobromus*, *Penicillius* and *Ceratochloa*. Within this clade, only two *B. erectus* sequences (KM077291 and KP987398) have a close relationship among them (98 PP-BI), whereas the others, including our *B. erectus* (br1) sample, form separated terminal branches. Similarly to our *B. picoeuropeanus* samples, four of the *B. picoeuropeanus* samples (Br2-6) plus some of the *B. erectus* samples collected in Picos de Europa (KP987399) grouped in the well-supported clade (99 BS-ML, 86 PP-BI), another formed a low-supported clade with *B. brachyantera* sequences (66 BS-ML, 57 PP-BI), and the rest of *B. picoeuropeanus* sequences form separated terminal branches.

**Figure 4:**
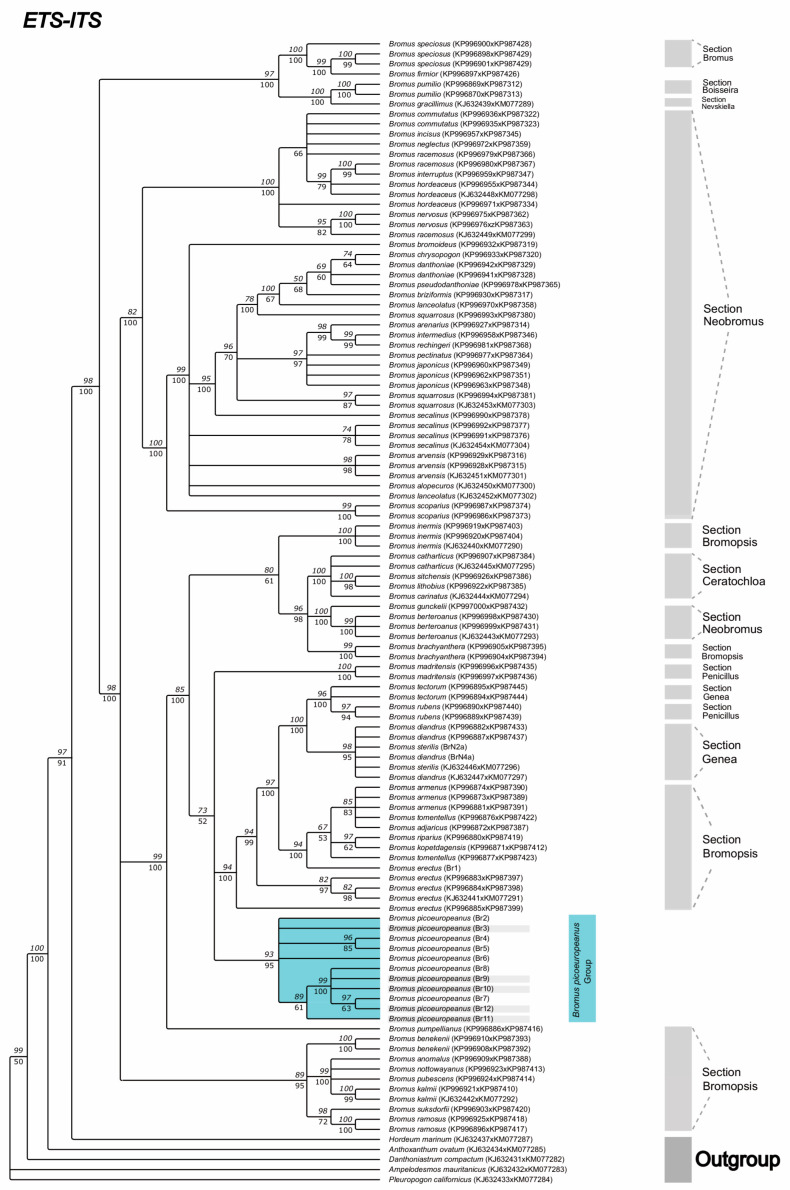
Consensus phylogenetic tree was obtained from ML and BI analyses based on the combination of the nuclear sequences ETS-ITS. The numbers over the branches correspond to the bootstrap (BS) values from the ML analysis, whereas the numbers under the branches represent the posterior probabilities (PP) obtained during the BI analysis. Br: new *Bromus* samples were generated in this study. The Br samples from the Cantabrian Mountains and from Picos de Europa have been highlighted in blue. The Br sample from lower lands has been highlighted in gray.

Analysis of the ETS-ITS alignment (see Figure 4) presented a Cantabrian Mountains *B. picoeuropeanus* exclusive clade with high branch support (93 BS-ML, 95 PP-BI), which included the individuals sampled outside the putative altitudinal range of *B. picoeuropeanus* (Br2 and Br9-12). Within this exclusive clade, the individuals sampled in the northmost locations, which belong almost outside (circa 1600 m) (Br7-8) or outside the altitudinal putative range of *B. picoeuropeanus* (Br9-12), formed a high to moderately supported subclade (89 BS-ML, 61 PP-BI).

This *B. picoeuropeanus* clade had a weak to moderately supported sister relationship (73 BS-ML, 52 PP-BI) with the *B. madritensis* clade (100 BS-ML, 100 PP-BI), as well as the clade comprising the *B. erectus* samples (including Br1) (94 BS-ML, 100 PP-BI) and the rest of sequences with the large indel. In this former clade, although all the *B. erectus* sequences were present, they did not group in an exclusive subclade. *B. erectus* Br1 sequence had a close phylogenetic relationship with the subclade formed by *B. tomentellus*, *B. riparius*, *B. kopetdagensis*, *B. armenus* and *B. adjaricus* (94 BS-ML, 100 PP-BI), while 3 *B. erectus* samples formed a small clade (82 BS-ML, 97 PP-BI) and another formed a separated terminal branch.

**Figure 5:**
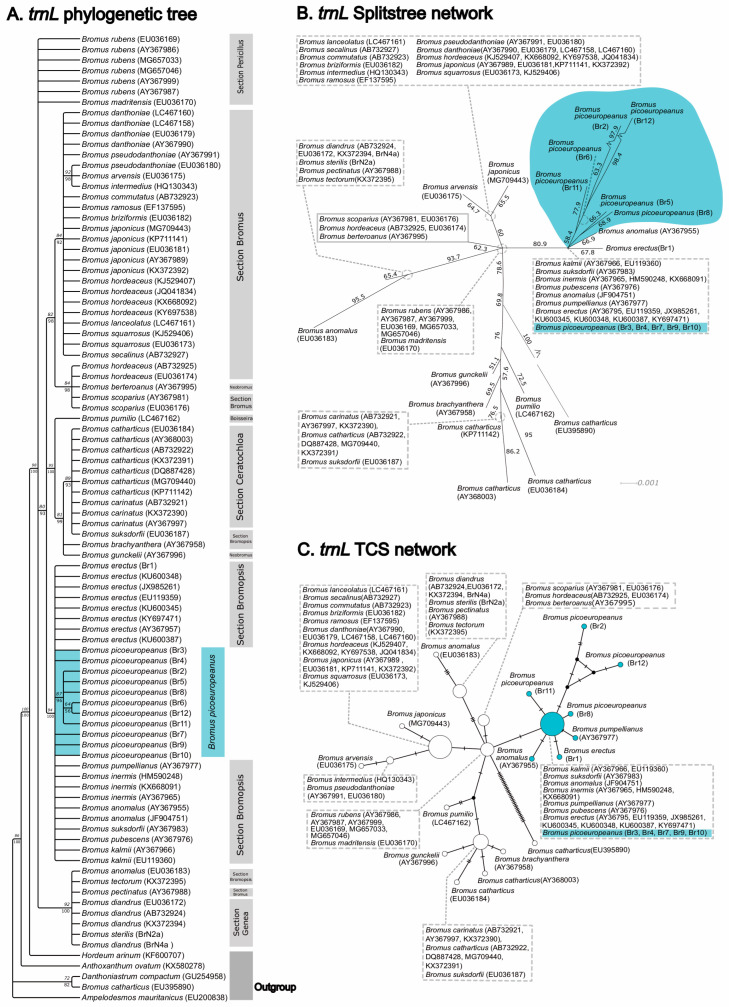
Phylogenetic and network analyses based on trnL sequences. (**A**) Consensus phylogenetic tree was obtained from the ML and BI analyses based on the plastid marker trnL. The numbers over the branches correspond to the bootstrap (BS) values from the ML analysis. The posterior probabilities (PP) were obtained during the BI analysis, whereas the numbers under the branches represent the posterior probabilities (PP) obtained during the BI analysis. Br: new *Bromus* samples were employed in this study. The Br samples from the Cantabrian Mountains and from Picos de Europa have been highlighted in blue. The Br samples from lower lands has been highlighted in gray. (**B**) Phylogenetic network generated by SplitsTree based on the trnLB dataset. Numbers near the branches represent bootstrap support values (BS). (**C**) TCS network obtained based on the trnLB dataset. Br: *Bromus* samples were generated in this study.

On the other hand, the phylogenetic analyses based on the plastid dataset (trnL) retrieved a topology in which independent phylogenetic analysis (see Figure 5A) retrieved a topology in which all the *B. picoeuropeanus* and *B. erectus* samples formed a well-supported clade (94 BS-ML, 100 PP-BI) together with other species of section *Bromopsis*. The clade was sister to two other clades, one formed mainly by species of section *Ceratochloa* (95 BS-ML, 100 PP-BI) and another formed mainly by species of section *Bromus* (82 BS-ML, 98 PP-BI). These results were further supported by the topologies of the splitstrees and TCS haplotype networks based on trnL (Figure 5B,C), in which all the *B. picoeuropeanus* and *B. erectus* samples formed a group with *Bromus* species belonging to section *Bromopsis* with high branch support (80.9 BS-splitstree network). Interestingly, some of the Cantabrian Mountains *B. picoeuropeanus* samples, including samples from Picos de Europa and their surrounding areas (Br2, Br5, Br6, Br8, Br11 and Br12), formed their own highly supported subclade in the tree analyses (87 BS-ML, 96 PP-BI) and also formed their own branches beyond the group including *B. picoeuropeanus* and *B. erectus* in the SplitsTrees and TCS haplotype networks.

**Figure 6:**
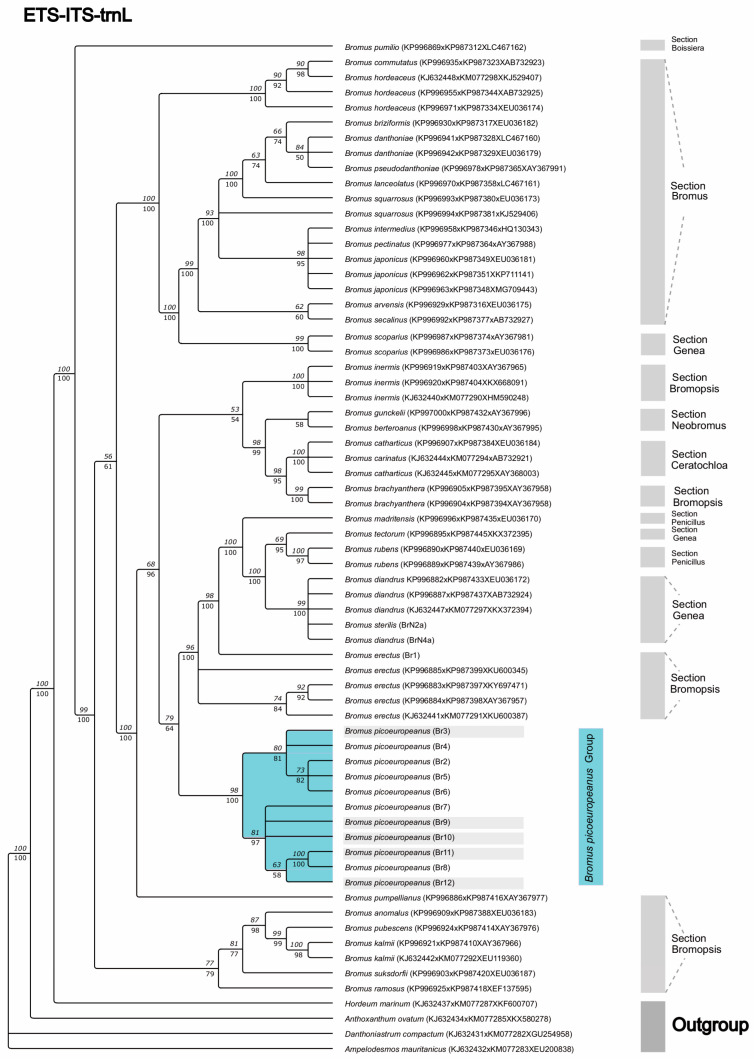
Consensus phylogenetic tree was obtained from ML and BI analyses based on the nuclear and chloroplastic combined sequences ETS-ITS-trnL (UAA). The numbers over and under the branches correspond to the branch support values. The numbers over the branches represent the bootstrap (BS) values obtained from the ML analysis, while the numbers under the branches correspond to the BI analysis posterior probability (PP) values. Br: *Bromus* samples were generated in this study. The Br samples from the Cantabrian Mountains and from Picos de Europa have been highlighted in blue. The Br samples collected under 1600 m above sea level has been highlighted in grey.

The topology of the ETS-ITS-trnL combined dataset (see Figure 6) presents a well-supported *B. picoeuropeanus* (2-12-Br) exclusive clade containing the Cantabrian Mountains and Picos de Europa samples (98 BS-ML, 100 PP-BI). This clade was subdivided in two subclades: one highly supported subclade (81 BS-ML, 97 PP-BI) comprising individuals from the northmost sampled locations which belong almost outside (circa 1600 m) or outside the altitudinal putative range of *B. picoeuropeanus* (Br7-12), another highly supported subclade (80 BS-ML, 81 PP-BI) formed by individuals sampled within the altitudinal range (Br4-Br6), and another individual outside the altitudinal range sampled in the southmost location (Br2). This clade is sister (79 BS-ML, 64 PP-BI) to another, which aggregated *B. erectus*, *B. diandrus*, *B. sterilis*, *B. rubens*, *B. tectorum* and *B. madritensis* sequences with high support (96 BS-ML, 100 PP-BI). Interestingly, in these analyses, the sequences of *B. erectus* did not generate an exclusive subclade, although three sequences formed a subclade with high statistic support (84 BS-ML, 88 PP-BI). This *B. erectus*, *B. diandrus*, *B. sterilis*, *B. rubens*, *B. tectorum* and *B. madritensis* clade belonged to a major clade (100 BS-ML, 100 PP-BI), which included a low-supported clade (53 BS-ML, 54 PP-BI) comprising species from sections *Bromopsis* (*B. branchyanthera* Döll and *B. inermis* Steven), *Ceratochloa* (*B. catharticus* Vahl and *B. carinatus* Hook. and Arn.), *Neobromus* (*B. berteroanus* Colla and *B. gunckelii* Matthei) and *Genea* (*B. diandrus* and *B. sterilis*) and a separate terminal branch formed by *B. pumpellianus* Scribn (68 BS-ML, 96 PP-BI).

## 3. Discussion

The phylogenetic analyses based on ITS showed a topology in which the samples collected in the Cantabrian Mountains (Br2-12) were polyphyletic as they formed part of various clades or separate terminal branches of the same clade. However, the same analyses based on ETS generated an exclusive *B. picoeuropeanus* clade formed by all the individuals collected in the Cantabrian Mountains, including those outside the putative altitudinal range of this species as provided by Acedo and Llamas [29]. The combination of both nuclear markers (ETS-ITS) also showed a similar topology in which the *Bromus* of the Cantabrian mountains also formed an exclusive monophyletic clade separated and sister to that comprising *B. erectus* individuals. These results suggest that the *Bromus* collected in the Cantabrian Mountains would be a separate entity from *B. erectus*, thus discarding Hypothesis 4. The phylogenetic tree based on nuclear markers plus the plastid marker trnL further clarified these results, as the relationships of the obtained clades presented higher support values. On the other hand, the plastid marker was incapable of group taxa in species-exclusive clades. The trees and networks based on trnL presented large groups formed by many different species, with *B. erectus* and *B. picoeuropeanus* found in the section *Bromopsis* group. These results are similar to those of Nasiri et al. [51], who used a similar methodology to study the phylogenetic relationship within sect. *Bromus* and determined that neither the ITS nor the plastid markers were capable of discriminating at the species level, while the combination of ITS and ETS allowed them to be discriminated at species level.

The phylogenetic analyses strongly suggest that *Bromus picoeuropeanus* is molecularly distinct from *B. erectus* s. s., especially since the former did not form a subclade within a *B. erectus* s. s., exclusive clade from the ETS, ETS-ITS and ETS-ITS-trnL topologies. This phylogenetic position supports in part the hypothesis of Acedo and Llamas [29] who defined *B. picoeuropeanus* as an independent species based on morphological and ecological data. Therefore, the phylogenetic position results would support our Hypotheses 1, 2, 3 and 5, as all of them assume that both taxa are different species. Nevertheless, the altitudinal distribution of the sampled individuals neither supports the distribution or habitat proposed by Acedo and Llamas [29] by those two species in the study area (Hypothesis 2) nor the hypothesis of a sympatric distribution of the two species (Hypothesis 3). The presence of *B. picoeuropeanus* would not be restricted to Picos de Europa, as the samples collected in other locations of the Cantabrian Mountains also formed part of the *B. picoeuropeanus* clade, therefore indicating that *B. picoeuropeanus* would be a Cantabrian Mountains endemism rather than a Picos de Europa endemism.

Our results also shed some light on the altitudinal range of distribution of this endemism, since we found *B. picoeuropeanus* individuals occurring at altitudes ranging from at least 729 to 2200 m, a range wider than first supposed by Acedo and Llamas [29]. This indicates that the altitudinal occurrence might be wider at the lower altitudes than first thought by Acedo and Llamas [29], thus supporting our Hypothesis 5 regarding *B. picoeuropeanus* in Picos de Europa, namely that it is a different species from *B. erectus* s. s., the only one that inhabits the area. Interestingly, the samples from the south of the Cantabrian Mountains, where *B. erectus* s. s. has been reported to occur at height ranging from 1490 to 1520 m, Acedo and Llamas [13] was observed to belong to the *B. picoeuropeanus* clade. Although these sample were collected at lower and higher heights than that provided by Acedo and Llamas [13], these findings cast doubts regarding the actual distribution range of *B. erectus* s. s. in the south of the Cantabrian Mountains, as by the time this distribution was considered, *B. picoeuropeanus* had not yet been described. Biogeographically, we also determined that the two subclades within the *B. picoeuropeanus* subclade generated in the combined nuclear and plastid analyses separated *B. picoeuropeanus* individuals following bioclimes. On the one hand, we detect the highly supported subclade containing the *B. picoeuropeanus* individuals collected in Picos de Europa at heights ranging from 728 to 1653 m, which would belong to the lower altitudes of the orotemperate bioclime, while on the other hand, we have the other subclade containing samples found at subalpine regions of the orotemperate bioclime (at Picos de Europa from 1841 to 1928 m) plus the samples collected in the south of the Cantabrian Mountains, which belongs to the Orosubmediterranean (León sample) and to the supramediterranean bioclimes.

Another compelling result from our analyses is that *B. picoeuropeanus* belongs to a major clade that grouped together species from various sections with different ploidy levels, for instance *B. diandrus*, *B. inermis* and *B. carinatus* (8x), *B. erectus* and *B. tectorum* (4x), *B. catharticus* (6x), *B. sterilis* and *B. rubens* (2x) [13,52,53]. This suggests that the ploidy level of *B. picoeuropeanus* should be investigated to provide a better understanding of its phylogenetic relationships.

Regarding the relationship of *B. picoeuropeanus* with *B. erectus*, they seem to be closely related as in the combined nuclear plastid analyses *B. picoeuropeanus* is a sister to the subclade comprising *B. erectus* (section *Bromopsis*), *B. rubens* (section *Penicillius*), *B. diandrus* and *B. tectorum* (section *Genea*). Nevertheless, our molecular evidence based on nuclear and plastid makers, separately and in combination, do not allow to determine the section in which *B. picoeuropeanus* should be placed, as either of the *B. picoeuropeanus* species exclusive clade belonged to clades formed by species from different sections, or members of the *Bromopsis* section were located in various clades. Hence, our evidence does not entirely support the proposal by Rico and Acedo [54] regarding the section *Pnigma* for *B. picoeuropeanus*. On the other hand, the described *Bromus* section *Penicillus* [29], which comprises *B. madritensis*, *B. rubens* and *B. fasciculatus*, is not supported as a monophyletic group by molecular evidence.

The ITS and ETS analyses portrayed *B. erectus* as a polyphyletic group, whereas the nuclear plastid analyses generated topologies in which three *B. erectus* samples generated a monophyletic clade in which the *Bromus* sample collected at lower altitudes (Br1), which corresponded to the description of *B. erectus*, was not included. This sample (Br1) belonged to the same subclade as *B. erectus*, although its relationship was closer to the species from section *Genea B. diandrus*, *B. rubens* and *B. tectorum*. The nuclear-combined analyses also indicated a closer relationship with *B. armenus* Boiss., *B. adjaricus* Sommier and Levier, *B. riparius* Rehmann, *B. kopetdagensis* Drobow and *B. tomentellus* Boiss. These differences in phylogenetic relationships could be explained by the fact that the combined nuclear analyses had more sequences than the combined analysis with trnL due to the lower availability of trnL sequences. Hence, although the latter analyses generated more reliable relationships, the former presented more reliable potential relationships for *B. erectus* samples. Therefore, our results cast doubts regarding the adscription of the individual Br1 as *B. erectus* s. s., since the morphological differentiation provided by Acedo and Llamas [29] included several *B. erectus* vouchers from different European herbaria. This means that our sample identified as *B. erectus* (Br1) could not be *B. erectus* s. s. This hypothesis would be in accordance with the position Br1 in the ETS-ITS and ETS-ITS-trnL topologies with respect to the rest of the individuals identified as *B. erectus*. In this sense, we cannot determine whether our sample Br1 is the one that does not belong to *B. erectus* s. s. or whether the other samples from previous studies are the ones that do not correspond, as no sample from the type locality has been sequenced yet (see Table S1). This situation has wider implications, affecting *B. picoeuropeanus* and other species morphologically similar to *B. erectus* as well. Therefore, our evidence, given the current absence of *B. erectus* type locality samples, only allow us to state that the individuals belonging to *B. picoeuropeanus* do not belong to the same taxon as the individuals known as *B. erectus* in our study area.

The altitudinal distribution of the taxon known as *B. erectus* in Picos de Europa and its surrounding areas seems to be more similar to that provided by Bačič and Jogan [38] for the *B. erectus* s. s. in the Alps, which considered 600 m its distributional limit, although our sample was collected in even lower lands (312 m). All this evidence suggests that a better understanding of the altitudinal distribution of *B. erectus* could be vital in understanding the evolutionary history of this species and its relationship with morphologically similar species, such as *B. picoeuropeanus* in the Cantabrian Mountains or *B. transylvanicus* and *B. condensatus* in the Alps [38]. This perspective seems interesting taking into account the fact that Rico and Acedo [54] estimate that there are around 30 endemic taxa that have been reported to have a similar relationship to that of *B. picoeuropeanus* and *B. erectus* in Europe and the Mediterranean basin. In the actual context, our knowledge of the *B. erectus* complex and the genus *Bromus* would benefit from wider studies focusing on (1) detecting the microspecies currently included in this complex, (2) determining their distributions and (3) understanding ecological and evolutionary processes involved in the formation of these species. These types of studies would also be of importance in detecting potential morphological adaptations in *Bromus*, which could clarify whether the species of *B. erectus* complex are phylogenetically related or whether their similar morphology is due to convergence.

On the other hand, more information about *B. picoeuropeanus* is needed, since its mountain distribution indicates that this species is susceptible to experiencing range shifts as consequence of temperature rise as has already been reported in other *Bromus* species [55]. In the context of climate change, its capacity will depend on many factors, such as the genetic structure of the existing population [56]; therefore, future research efforts should focus on these conservational aspects.

## 4. Materials and Methods

### 4.1. Plant Material

**Figure 7:**
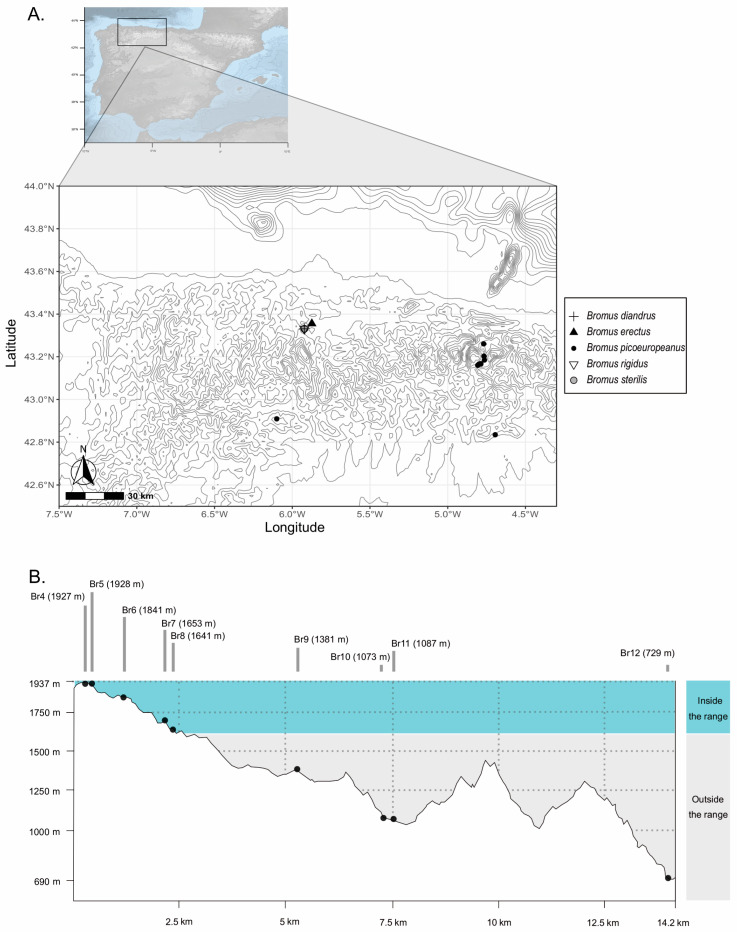
(**A**) Sampling area of the *Bromus* samples of this study, comprising collations in the Principality of Asturias, Cantabria, León and Palencia. The black dots (individuals Br2-12) and the black triangle (individual Br1) represent the sampled individuals of the *B. erectus* complex (i.e., *B. erectus* s. l.), while the cross, the white triangle and the grey dots represent the *B. diandrus*, *B. sterilis* and *B. rigidus* individuals, respectively. (**B**) Vertical profile of the clinal sampling of *Bromus picoeuropeanus* samples (Br2-12) in Picos de Europa mountainous area. The altitudes (measured in meters above sea level (m. a. s. l.) inside the *B. picoeuropeanus* range as defined by Acedo and Llamas [29] have been highlighted in blue, while altitudes outside this range have been highlighted in gray.

A total of twelve individuals of *B. erectus* s. l. (i.e., *B. erectus* complex) were collected in Picos de Europa and other regions of the Cantabrian Mountains (see Figure 7A). Nine of those individuals were collected at different sites of Picos de Europa and their surrounding areas, following a clinal sampling scheme in which heights comprised altitudes ranging from 729 to 1928 m (see Figure 7B and Table 2), thus including the altitude ranges of *B. erectus* s. s. and *B. picoeuropeanus* described by Acedo and Llamas [29] for this area—from 1600 to 2200 m for *B. picoeuropeanus* and from 1490 to 1520 m for *B. erectus* s. s. Additionally, other two individuals of *B. erectus* complex, one fitting in the range of *B. erectus* s. s. and another fitting the range of *B. picoeuropeanus* as proposed by Acedo and Llamas [29], were sampled at locations in the Cantabrian Mountains outside the limits of Picos de Europa. Finally, an individual of *B. erectus* complex fitting the altitudinal range for *B. erectus* s. s. proposed by Bačič and Jogan [38]—up to 600 m a. s. l.—was sampled on the north side of the Cantabrian Mountains to serve as contrast from individuals identified as *B. picoeuropeanus* collected within the range of *B. erectus* s. s. sensu Acedo and Llamas [29].

This sampling design resulted in a total of six sites fitting within the altitudinal range of *B. erectus* s. s. sensu Acedo and Llamas [29], which also fitted its area of distribution of the north of Spain provided by Acedo and Llamas [29] and Rico and Acedo [54]: Br1, the only location fitting the distribution proposed by Bačič and Jogan [38], Br3 and Br9-12. The number of sites fitting the *B. picoeuropeanus* altitudinal range sensu Acedo and Llamas [29] was six (Br2 and Br4-Br8). All the collected individuals were later identified as either *B. erectus* s. s. or *B. picoeuropeanus* following the detailed comparative of Acedo and Llamas [29] (see Table 2).

Finally, three individuals belonging to *B. diandrus* Roth (1787), *B. sterilis* L. (1753) and *B. rigidus* Roth (1790), which form part of different sections from that of *B. picoeuropeanus* and *B. erectus* s. s., were identified following Rico and Acedo [54] and Smith [57] and collected (see Figure 7A). The collected material consisted of complete individuals, which were preserved in silica gel before the DNA extraction.

### 4.2. DNA Extraction, Amplification and Sequencing

The DNA extraction was conducted using the NucleoSpin^®^ Plant II Columns (Macherey-Nagel, GmbH & Co. KG, Düren, Germany) kit. The extracted DNA was stored at −20 °C. Three molecular markers which had proved useful in previous studies of *Bromus* were amplified: the two high-copy nuclear markers Internal Transcribed Spacer (ITS) and External Transcribed Spacer (ETS) and the plastid marker trnL. The regions 5.8S, ITS-1, and ITS-2 of the ribosomal nuclear maker ITS were amplified by PCR using the primers 17SE and 26SE [58]. The partial sequence 3′ETS of the intergenetic spacer (IGS) was amplified using the primers RETS-B4F [59] and 18S-R [60] following the recommended PCR conditions and cycles of Alonso et al. [59]. The exon of chloroplastic sequence trnL was amplified with the c and d pair of primers [61], following their proposed PCR conditions and the PCR cycle. Obtained PCR products were sequenced at the DNA Synthesis and Sequencing Facility Macrogen (Amsterdam, The Netherlands).

### 4.3. Phylogenetic Analysis

The obtained sequences were visualized and manually edited in Geneious Prime v. 1.3 [62]. The bases and polymorphism were coded following the International Union of Pure and Applied Chemistry (IUPAC). SNPs were considered “true” if they occurred at the same site in both reverse and forward amplicons and the lower peak reached at least a third of the height of the higher one.

Since previous molecular studies have revealed that the section *Bropmosis*, in which *B. picoeuropeanus* was classified by Rico and Acedo [54], is polyphyletic e.g., [14,51], sequences from different *Bromus* sections generated in previous studies were included in the analyses. The newly generated sequences together with *Bromus* sequences from previous studies available at GenBank (see Table S1) were used to generate several datasets: the ITS dataset, ETS dataset, trnL dataset, nuclear dataset, and the combined dataset. The nuclear dataset consisted of the concatenation of ETS and ITS sequences, while the combined dataset was formed by the concatenation of all three markers. We only included *Bromus* species from which sequences of all three makers were available; these included the type of the genus *Bromus secalinus* L. (1753). When generating the combined datasets, we tried to concatenate sequences obtained from the same voucher whenever possible. The outgroups of the analyses were *Pleuropogon californicus* (Nees) Benth. (1883), *Hordeum marinum* Huds. (1778), *Danthoniastrum compactum* (Boiss. and Heldr.) (1970), *Ampelodesmos mauritanicus* (Poir.) T.Durand and Schinz (1894) and *Anthoxanthum ovatum* Lag. (1816) since they have been used in previous studies of this genus [59,63].

The sequences were aligned in MUSCLE [64] using the online server EMBL-EBI [65] and the alignment was manually reviewed and edited in Geneious Prime v.1.3 [66] The nucleotide substitution model of each dataset was estimated in JModelTest 2 v.1.10 [67] by the default setting of the corrected Akaike Information Criterion (AIC) [68]. In the case of the ITS and ETS datasets, the inferred substitution model was the symmetrical substitution model with gamma distribution (SYM + G) [69], while the substitution model estimated for the chloroplastic sequence trnL (UAA) was the Hasegawa–Kishino–Yano substitution model with gamma distribution (HKY + G) [70].

The phylogenetic relationships of the samples were inferred by two different phylogenetic methods: Maximum Likelihood (ML) and Bayesian Inference (BI). The ML analysis was conducted in the IQ-TREE web service [71,72]. For this analysis, the initial tree was estimated by Neighbor-Joining (NJ) and the posterior full-tree rearrangement operations were performed by Neighbor Interchange (NNI). The branch support values were statistically inferred by 10,000 bootstrap (BS) replications [73,74,75]. The BI inference using MrBayes [76] was performed by 6 Monte Carlo Markov Chains (MCMC) (1 cold chain and 5 hot chains) for 10,000,000 generations and a 0.25 of burnin fraction. This burnin fraction was visualized using Tracer v1.7.1 [77]. The branch support was statistically inferred by posterior probability (PP).

The three phylogenetic analyses were performed on each maker (ETS, ITS, and trnL (UAA)) and combined datasets (the nuclear ETS-ITS and the plastid plus nuclear markers). In the cases where the sequences were concatenated, the ML and BI analyses were performed using a partitioned analysis and applying the corresponding model. Since a large indel was found in the ETS dataset, we performed and compared analyses with and without the insertion, finding similar topologies with similar support values. For this reason, we used the ETS dataset containing the large indel to perform the phylogenetic analyses.

We also inferred the phylogenetic relationships through the generation of phylogenetic networks based on the plastid haplotype diversity, hence we generated a second trnL dataset (trnLB) which only included taxa belonging to *Bromus*. The best-fitting substitution model for this dataset was HKY + G [70]. On the one hand, we constructed a Neighbournet in SplitsTree 4.16.2 [78], estimating branch support by performing 10,000 bootstrap repetitions. On the other hand, we also generated a gene genealogy by Templeton, Crandall and Sing (TCS) cladistics methods [79] in PopART 1.7 [80].

## Figures and Tables

**Table 1:** Main features of the different alignments used in the phylogenetic analysis and the haplotype network. The number of analyzed taxa, the conservative sites, the variable sites, and the parsimonious-informative sites refers only to *Bromus* taxa. The number of sequences includes the outgroups, while the rest of the features does not include the outgroups. Variable sites are sites in which a minimum of two different nucleotides occur, while parsimonious-informative sites are those in which a minimum of two different nucleotides occur, two of which must have a minimum frequency of two. The ranges of length of the sequences and the alignment length were measured in pairs of bases (pb).

	ETS	ITS	trnL	Nuclear	Combined
*Bromus* analyzed taxa	51	50	34	51	34
Number of sequences	119	150	87	109	63
Range of length of sequences (pb)	160–497	512–540	425–465	691–1034	1139–1490
Alignment length (pb)	529	568	508	1088	1678
C + G (%)	52.7	57.8	28.7	55.6	46.8
Conserved sites	300	377	413	684	1196
Variable sites	214	169	80	375	337
Parsimonious-informative sites	150	123	22	272	191

**Table 2:** Code of the samples used in this study, location of the sampled populations (coordinates), voucher, collector and identifier, the morphological identification flowing the given references and GenBank accession numbers. JAFP (=José Antonio Fernández Prieto), HSN (=Hermino S. Nava).

Code	Location	Coordinates	Altitude (m a. s. l.)	Voucher	Collector (Identifier)	Morphological Identification	GenBank Accession n°
Br1	Faculty of Biology, Oviedo (Asturias)	43°21′19.90″ N, 5°52′26.85″ W	312	FCO40796	JAFP and HSN (HSN)	*B. erectus*	ETS: OQ557418 ITS: OQ544412 trnL: OQ557422
Br2	Riolago de Babia, Babia, León (Castilla y León)	42°54′32.39″ N, 6°5′59.88″ W	1693	FCO40799	JAFP and HSN (HSN)	*B. picoeuropeanus*	ETS: OQ557413 ITS: OQ544413 trnL: OQ557428
Br3	Peña Grande, Villaverde de la Peña, Palencia (Castilla y León)	42°50′44.36″ N, 4°41′40.59″ W	1625	FCO40800	JAFP and HSN (HSN)	*B. picoeuropeanus*	ETS: OQ557408 ITS: OQ544414 trnL: OQ557423
Br4	Picos de Europa National Park (Cantabria)	43°9′38.21″ N, 4°48′23.65″ W	1927	FCO40801	JAFP and HSN (HSN)	*B. picoeuropeanus*	ETS: OQ557412 ITS: OQ544418 trnL: OQ557424
Br5	Picos de Europa National Park (Cantabria)	43°9′37.32″ N, 4°48′24.64″ W	1928	FCO40802	JAFP and HSN (HSN)	*B. picoeuropeanus*	ETS: OQ557416 ITS: OQ544411 trnL: OQ557429
Br6	Picos de Europa National Park (Cantabria)	43°9′56.55″ N, 4°47′57.18″ W	1841	FCO40803	JAFP and HSN (HSN)	*B. picoeuropeanus*	ETS: OQ557414 ITS: OQ544409 trnL: OQ557430
Br7	Picos de Europa National Park (Cantabria)	43°10′3.82″ N, 4°47′17.22″ W	1653	FCO40804	JAFP and HSN (HSN)	*B. picoeuropeanus*	ETS: OQ557411 ITS: OQ544406 trnL: OQ557425
Br8	Picos de Europa National Park (Cantabria)	43°9′57.50″ N, 4°47′13.05″ W	1641	FCO40805	JAFP and HSN (HSN)	*B. picoeuropeanus*	ETS: OQ557417 ITS: OQ544407 trnL: OQ557427
Br9	Picos de Europa National Park (Cantabria)	43°11′5.31″ N, 4°45′50.11″ W	1381	FCO40806	JAFP and HSN (HSN)	*B. picoeuropeanus*	ETS: OQ557409 ITS: OQ544410 trnL: OQ557431
Br10	Picos de Europa National Park (Asturias)	43°12′11.19″ N, 4°46′1.71″ W	1073	FCO40807	JAFP and HSN (HSN)	*B. picoeuropeanus*	ETS: OQ557415 ITS: OQ544408 trnL: OQ557426
Br11	Picos de Europa National Park (Asturias)	43°12′7.17″ N, 4°46′0.00″ W	1087	FCO40808	JAFP and HSN (HSN)	*B. picoeuropeanus*	ETS: OQ557407 ITS: OQ544419 trnL: OQ557432
Br12	Picos de Europa National Park (Asturias)	43°15′38.29″ N, 4°46′8.46″ W	729	FCO40809	JAFP and HSN (HSN)	*B. picoeuropeanus*	ETS: OQ557410 ITS: OQ544415 trnL: OQ557433
BrN2a	Las Caldas, Oviedo (Asturias)	43°19′52.13″ N, 5°55′20.338″ W	99	FCO40811	HSN (HSN)	*B. sterilis*	ETS: OQ557420 ITS: OQ544416 trnL: OQ557434
BrN3a	Las Caldas, Oviedo (Asturias)	43°19′52.13″ N, 5°55′20.338″ W	99	FCO40813	HSN (HSN)	*B. rigidus*	ETS: OQ557421 ITS: n.d. trnL: n.d.
BrN4a	Las Caldas, Oviedo (Asturias)	43°19′52.13″ N, 5°55′20.338″ W	99	FCO40814	HSN (HSN)	*B. diandrus*	ETS: OQ557419 ITS: OQ544417 trnL: OQ557435

## Data Availability

The data that support the findings of this study are openly available in GenBank at https://www.ncbi.nlm.nih.gov/genbank/ (accessed on 16 March 2023). The reference number of each sequence are specified in Table 2.

## Supplementary Materials

The following supporting information can be downloaded at: https://www.mdpi.com/article/10.3390/plants12071531/s1, Figure S1: Consensus phylogenetic tree obtained from the BI analyses based on the nuclear marker ETS without the large indel.; Table S1: List of sequences from *Danthoniastrum compactum*, *Ampelodesmos mauritanicus*, *Anthoxanthum ovatum*, *Pleuropogon californicus*, *Hordeum marinum* and *Bromus* used in our phylogenetic analyses. Refs [14,52,59,81,82,83,84,85,86,87,88,89,90,91,92,93,94,95,96,97,98] cited in Supplementary Materials.
