# Supplementary material for: What Hides in the Heights? The Case of the Iberian Endemism Bromus picoeuropeanus
Source: Plants (Basel). 2023 Apr 1;12(7):1531. doi: 10.3390/plants12071531 (PMC10096826; doi:10.3390/plants12071531)
Supplement: Supplementary file 1 [file plants-12-01531-s001.zip › plants-2209877-supplementary.pdf]

What hides in the heights? The case of the Iberian endemism *Bromus picoeuropeanus*

Claudia González-Toral [1], Herminio S. Nava [1], José Antonio Fernández Prieto [1,2] and Eduardo Cires[1,2*]

[1] Department of Organisms and Systems Biology, University of Oviedo, C/Catedrático Rodrigo Uría s/n, 33071 Oviedo, Spain.
[2] Institute of Natural Resources and Territorial Planning (INDUROT), Campus de Mieres, C/Gonzalo Gutiérrez Quirós s/n, 33600 Mieres, Spain.
* Correspondence: cireseduardo@uniovi.es; +0034104780

Table S1. List of sequences from *Danthoniastrum compactum, Ampelodesmos mauritanicus Anthoxanthum ovatum, Pleuropogon californicus, Hordeum marinum* and *Bromus* used in our phylogenetic analyses (Hsiao et al. 1995; Saarela et al. 2007; Cialdella et al. 2007; Barkworth et al. 2008; Schardl et al. 2008; Essi et al. 2008; Fortune et al. 2008; Bouchenak-Khelladi et al. 2009; Romaschenko et al. 2010; Refulio-Rodriguez et al. 2012; Wallinger et al. 2012; Hiiesalu et al. 2012; Mason-Gamer 2013; Matsushima et al. 2013; Alonso et al. 2014; Pimentel et al. 2017; Yao et al. 2017; Chumová et al. 2017; Holá et al. 2017; Kowalczyk et al. 2019; Pourmoshir et al. 2019). The gathered data associated to each sequence follow this order: taxon name, collection site, collector, voucher and GenBank accession numbers.

ETS:

Bromus adjaricus Sommier & Levier, Armenia, Aedo et al., Aedo et al., KP996872. ***B. alopecuros*** Poir., Tiznt, Jbel Imzi (Morocco) F. Llamas and F. Gomiz, LEB -11 (2004), KJ632450. ***B. anomalus*** Rupr. ex Fourn., Canda, Ledingham, Ledingham, KP996909. ***B. arenarius*** Labill., U.S.A, Ferris, Wiggins and Ernst 13906, KP996927. ***B. armenus*** Boiss., Turkey, Aedo et al., Aedo et al. AH1665, KP996874. ***B. armenus*** Boiss. Turkey, Aedo et al., Aedo et al. AH1691, KP996873. ***B. armenus*** Boiss. Turkey, Mutlu, Mutlu 918, KP996881. ***B. arvensis*** L., Botanischer Garten Oldenburg (Germany) 06-116-74-74 Botanischer Garten Oldenburg, A. Alonso, LEB-344 (2011) KJ632451. ***B. arvensis*** L., Spain, Acedo et al., Acedo et al. 232, KP996929. ***B. arvensis*** L., Spain, Acedo et al., Acedo et al. 232, KP996928. ***B. benekenii*** (Lange) Trimen, Austria, Kleesadl and Kupka, Kleesadl and Kupka, KP996910. ***B. benekenii*** (Lange) Trimen, Spain, Acedo et al., Acedo et al. 213, KP996908. ***B. berteroanus*** Colla, Raquia-Cajacay, Bolognesi (Peru), P.M. Peterson, MO-17689 (2005), KJ632443. ***B. berteroanus*** Colla, Chile, Kalin Arroyo, Maldonado and Diaz, Maldonado and Diaz 995015, KP996998. ***B. berteroanus*** Colla, Chile, Teillier, Teillier 5843, KP996999. ***B. brachyanthera*** Doll., Brazil, Longhi-Wagner, Longhi-Wagner 10882, KP996905. ***B. brachyanthera*** Doll., Brazil, Scur, KP996904. ***B. briziformis*** Fisch. & C.A.Mey, U.S.A., Williams, Williams 88281, Tiehm and Nachlinger, KP996931. ***B. briziformis*** Fisch. & C.A.Mey, Turkey, Gillespie et al., Gillespie et al., KP996930. ***B. bromoideus*** (Lej.) Crép., Belgium, Alonso, Alonso 349, KP996932. ***B. carinatus*** Hook. & Arn; California (USA), P.M. Peterson, CAN-19700 (2006), KJ632444. ***B. catharticus*** Vahl., Collanzo, Asturias (Spain), A. Alonso and R. Noriega, LEB-132 (2011), KJ632445. ***B. catharticus*** Vahl., Spain, Acedo, Acedo 92, KP996907. ***B. catharticus*** Vahl., Spain, Acedo, Acedo 23, KP996906. ***B. chrysopogon*** Viv., Iran, Acedo and Llamas, Acedo and Llamas 254, KP996933. ***B. commutatus*** Schrad., Spain, Acedo and Llamas, Llamas and Acedo 49, KP996936. ***B. commutatus*** Schrad., Germnay, Kress, Kress 1, KP996935. ***B. danthoniae*** Trin., Iran, H. Mirzaie-Nodoushan, H. Mirzaie-Nodoushan, 1, KP996940 ***B. danthoniae*** Trin., Iran, H. Mirzaie-Nodoushan, H. Mirzaie-Nodoushan 9, KP996942. ***B. danthoniae*** Trin., Iran, H. Mirzaie-Nodoushan, H. Mirzaie-Nodoushan 2, KP996941. ***B. danthoniae*** Trin., Turkey, Donmez, Murlu and Agar, Donmez 13325 Murlu and Agar, KP996939. ***B. danthoniae*** Trin., Turkey, Aedo et al., Aedo et al., KP996938. ***B. diandrus*** Roth, Beni Chiker, Nador (Morocco), J. Chrtek and Z. Doc˘kalova, LEB - (2010), KJ632447. ***B. diandrus*** Roth, Iran, Alonso, Alonso 347, KP996887. ***B. diandrus*** Roth, Spain, Acedo and Llamas, Acedo and Llamas 91, KP996882. ***B. erectus*** Huds., Picos de Europa-Macizo Oriental, Cantabria (Spain), C. Acedo, A. Alonso, and F. Llamas, LEB-247(2011), KJ632441. ***B. erectus*** Huds. Spain, Acedo, Acedo et al. 224, KP996885. ***B. erectus*** Huds., Spain, Acedo, Acedo 208, KP996884. ***B. erectus*** Huds., Spain, Alonso and Noriega, Alonso and Noriega 188, KP996883. ***B. firmior*** Stapf, South Africa, Panagos, Panagos 48, KP996897. ***B. gunckelii***

Mathei, Chile, Peterson, Peterson 15613, KP997000. ***B. hordeaceus*** L., Calera de León, Badajoz (Spain), C. Acedo, F.M. Vazquez, A. Alonso and F. Llamas, LEB-109 (2011), KJ632448. ***B. hordeaceus*** L., Spain, Llamas, Acedo and Alonso 15, KP996956. ***B. hordeaceus*** L, Spain, Gutierrez and Vazquez, Gutierrez and Vazquez, KP996955. ***B. hordeaceus*** L., Germnay, Gregor, Gregor 3515, KP996971. ***B. incisus*** R.Otto & H.Scholz,, Germany, Otto, Otto, KP996957. ***Bromus inermis*** Leyss. Kyakhtinskii Raion, Buryatiya Republic (Russia), V. Chepinoga, LEB-28355 (2010), KJ632440. ***Bromus inermis*** Leyss, Russia, Chepinoga, Chepinoga, Bi2, KP996920. ***Bromus inermis*** Leyss, Russia, Chepinoga, Chepinoga, Bi2, KP996919. ***B. intermedius*** Guss, Turkey, Donmez, Donmez 3350, KP996958. ***B. interruptus*** Duce, England, KP996959. ***B. japonicus*** Thunb., Hungary, Chrtek and Dockalova, Chrtek and Dockalova 18/1, KP996963. ***B. japonicus*** Thunb., Turkey, Aedo et al., Aedo et al. CA6570, KP996962. ***B. japonicus*** Thunb.,Turkey, Donmez, Donmez 3371, KP996960. ***B. kalmii*** A. Gray, Everwilde Farm (USA), C. Acedo, LEB-332 (2013), KJ632442. ***B. kalmii*** A. Gray, Canada, M.J. Oldham and S.R. Brinker 37463, KP996921. ***B. kopetdagensis*** Drobow, Iran, H. Mirzaie-Nodoushan, H. Mirzaie-Nodoushan 7, KP996871. ***B. lanceolatus*** Roth., Ribera de la Albuera, Badajoz (Spain), C. Acedo, F.M. Vazquez, A. Alonso and F. Llamas, LEB-101 (2011), KJ632452. ***B. lanceolatus*** Roth., Armenia, Aedo et al., Aedo et al., KP996970. ***B. lanceolatus*** Roth, Spain, Leon, Losada and Sosa, Leon, Losada and Sosa 2, KP996968. ***B. lithobius*** Trin., Chile, Carrasco-Falrias, Carrasco-Falrias 2, KP996922. ***B. madritensis*** L., Spain, Acedo, Alonso and Llamas, Acedo, Alonso and Llamas 99, KP996997. ***B. madritensis*** L., Spain, Acedo, Alonso and Llamas, Acedo, Alonso and Llamas 99, KP996996. ***B. natalensis*** Stapf, South Africa, Saayman, Saayman 71, KP996899. ***B. neglectus*** Nyman, Greece, Hageman, Scholz and Scharz, Scholz and Scharz, KP996972. ***B. nervosus*** Acedo & Llamas, Spain, Garcia and Vazquez, Vazquez, KP996976. ***B. nervosus*** Acedo & Llamas, Portugal, Llamas, Acedo and Alonso, Llamas, Acedo and Alonso 9, KP996975. ***B. nottowayanus*** Fern., U.S.A., Rothfels, Rothfels 2873, KP996923. ***B. pectinatus*** Thunb., South Africa, Aizpuru et al., Aizpuru et al. LM6093, KP996977. ***B. pubescens*** Muhl. ex Willd., U.S.A., Peterson, Peterson 15788, KP996924. ***B. pumilio*** (Trin.)P.M.Sm., Turkey, Metzger, Metzger, KP996870. ***B. pumilio*** (Trin.)P.M.Sm., Armenia, Nersesyan, Nersesyan 50-2004, KP996869. ***B. pumpellianus*** Scribn., Russia, Chepinoga, Chepinoga Bp1, KP996886. ***B. pseudodanthoniae*** Drobov,, Iran, Alonso, Alonso 341, KP996978. ***B. racemosus*** Schrad, Cofinal, León (Spain), A. Alonso, LEB- 29 (2011), KJ632449. ***B. riparius***, Russia, Kiseleva, Kiseleva, KP996880. ***B. racemosus*** Schrad, Spain, Alonso, Alonso, Alonso 211, KP996980. ***B. racemosus*** Schrad, Spain, Acedo, Acedo 26, KP996979. ***B. ramosus*** Huds., Bulgaria, Aedo et al., Aedo et al., KP996925. ***B. ramosus*** Huds., Spain, Llamas et al. 79, KP996896. ***B. rechingeri*** Melderis, Iran, Alonso, Alonso 342, KP996981. ***B. rubens*** L., Spain, Aedo et al., Acedo et al. 106, KP996890. ***B. rubens*** L., Morocco, Chrtek and Dockalova, Chrtek and Dockalova, KP996889. ***B. scoparius*** L., Spain, Acedo, Alonso and Llamas; Acedo, Alonso and Llamas 94, KP996987. ***B. scoparius*** L., Portugal, Acedo, Alonso and Llamas; Llamas, Acedo and Alonso 8, KP996986. ***B. secalinus*** L., Botanischer Garten Oldenburg (accession 07-104-07-74) (Germany), A. Alonso, LEB-339 (2011), KJ632454. ***B. secalinus*** L., Germany, Alonso 345, KP996992. ***B. secalinus*** L., Russia, Shilova and Estarozhieva, Shilova and Estarozhieva, KP996991. ***B. secalinus*** L., Findland, Nurmi, Nurmi, KP996990. ***B. sitchensis*** Trin., U. S. A., Peterson, Peterson 19683, KP996926. ***B. speciosus*** Ness, South Africa, Catalan and Pimentel, Catalan and Pimentel SA043, KP996900. ***B. speciosus*** Ness, South Africa, Catalan and Pimentel SA043, KP996898. ***B. speciosus*** Ness, South Africa, Catalan and Pimentel, Catalan and Pimentel SA043, KP996901. ***B. speciosus*** Ness, Iran, H. Mirzaie-Nodoushan, H. Mirzaie-Nodoushan 1, KP996940. ***B. squarrosus*** L., Llombera, León (Spain), F. Llamas and C. Acedo, LEB-43 (2010), KJ632453. ***B. squarrosus*** L., Spain, Llamas, Llamas 187, KP996994. ***B. squarrosus*** L., Hungary, Chrtek and Dockalova, Chrtek and Dockalova 1, KP996993. ***B. suksdorfii*** Vasey, U. S. A., Cronquist, Cronquist, KP996903. ***B. suksdorfii*** Vasey, South Africa, Ellis, Ellis 5685, KP996902. ***B. sterilis*** L., Philistean Plain, Sou (Israel), A. Danin, T. Raus, W. Sauer, S. Brullo, B. Valdes, F. Amich, S.G. Gardner, R.C.H.J. van Ham, A. Gambino, F. Axelrod, Battia Pazy and Rivka Nokrian (1981), SALA, KJ632446. ***B. tectorum*** Huds., Iran, Alonso, Alonso 343, KP996895. ***B. tectorum*** Huds., Czech Republic, Chrtek, Chrtek 3, KP996894. ***B. tomentellus*** Boiss. Turkey, Oezuedogru and Agar, Oezuedogru and Agar, KP996876. ***B. tomentellus*** Boiss., Turkey, Doenmez, Doenmez, 2178 and Guener, KP996877. ***Ampelodesmos mauritanicus*** (Poir.) T. Durand & Schinz, Menorca, Balearic Islands, (Spain) Sa Mesquida, C. Acedo and F. Llamas; 253 (2011) LEB, KJ632432. ***Anthoxanthum ovatum*** Lag., Sardinia (Itaky) Iglesias M. Pimentel and E. Sahuquillo, (2009) SANT, KJ632434/KM077285. ***Danthoniastrum compactum*** (Boiss. & Heldr.) Holub, Mount Katarrachias, Thessaly (Greece), M. Röser, 10724 (2013) HAL KJ632431. ***Hordeum marinum*** Huds., Spain: Zamora, Villafafila, Zamora, Castilla y León, (Spain), C. Acedo and F. Llamas, 263 (2012) LEB, KJ632437. ***Pleuropogon californicus*** (Nees) Vasey, California (U. S. A.) Solano D. Sanchez Mata and M.G. Barbour, (1996) MA, KJ632433.

ITS:

Bromus adjaricus Sommier & Levier, Armenia, Aedo et al., Aedo et al., KP987387. ***B. alopecuros*** Poir., Tiznt, Jbel Imzi (Morocco) F. Llamas and F. Gomiz, LEB -11 (2004), KM077300. ***B. anomalus*** Rupr. ex Fourn. (1) PI 232199 (WRPIS) (USA), Keane 49 (ALTA), AY367905. ***B. anomalus*** Rupr. ex Fourn. (2) Tamaulipas (Mexico), Peterson 15918 & Valdes-Reyna (US), AY367906. ***B. anomalus*** Rupr. ex Fourn., Refulio-Rodriguez 145 (RSA), JF904806. ***B. anomalus*** Rupr. ex Fourn., Canda, Ledingham, Ledingham, KP987388. ***B. arenarius*** Labill., U.S.A, Ferris, Wiggins and Ernst 13906, KP996927. . ***B. armenus*** Boiss., Turkey, Aedo et al., Aedo et al. AH1665, KP987390. ***B. armenus*** Boiss. Turkey, Aedo et al., Aedo et al. AH1691, KP987389. ***B. armenus*** Boiss. Turkey, Mutlu, Mutlu 918, KP987391. ***B. armenus*** Boiss., Turkey, Aedo et al., Aedo et al. AH1665, KP987314. ***B. arvensis*** L. Botanischer Garten Oldenburg (Germany) 06-116-74-74 Botanischer Garten OldenburgA. Alonso, LEB-344 (2011), KM077301. ***B. arenarius*** Labill., U.S.A, Ferris, Wiggins and Ernst 13906, KP987314. ***B. armenus*** Boiss., Muzaffer Mukemre, Ilhan Kaya, MW262891. ***B. arvensis*** L., Spain, Acedo et al., Acedo et al. 232, KP987316. ***B. arvensis*** L., Spain, Acedo et al., Acedo et al. 232, KP987315. ***B. attenuatus*** Swallen, Tamaulipas and Nuevo León border (Mexico), Peterson 15926 & Valdes-Reyna (US), AY367910. ***B. benekenii*** (Lange) Trimen, U. S. A, Kleesadl and Kupka, Kleesadl and Kupka, KP987393. ***B. benekenii*** (Lange) Trimen, Spain, Acedo et al., Acedo et al. 213, KP987392. ***B. berteroanus*** Colla, PI 224789 (WRPIS) (Chile), Keane 37 (ALTA), AY367946. ***B. berteroanus*** Colla, Raquia-Cajacay, Bolognesi (Peru), P.M. Peterson, MO-17689 (2005), KM077293. ***B. berteroanus*** Colla, Chile, Kalin Arroyo, Maldonado and Diaz, Maldonado and Diaz 995015, KP987430. ***B. berteroanus*** Colla, Chile, Teillier, Teillier 5843, KP987431. ***B. brachyanthera*** Doll., La Paz (Bolivia), de Ros 9497 (US), AY367908. ***B. brachyanthera*** Doll., Brazil, Longhi-Wagner, Longhi-Wagner 10882, KP987395. ***B. brachyanthera*** Doll., Brazil, Scur, KP987394. ***B. briziformis*** Fisch. & C.A.Mey, U.S.A., Williams, Williams 88281, Tiehm and Nachlinger, KP996931. ***B. briziformis*** Fisch. & C.A.Mey, Turkey, Gillespie et al., Gillespie et al., KP987317. ***B. bromoideus*** (Lej.) Crép., Belgium, Alonso, Alonso 349, KP987319. ***B. carinatus*** Hook. & Arn., Durango (Mexico), Peterson 15421 et al. (US), AY367948. ***B. carinatus*** Hook. & Arn., South Korea, J H Lee, JHLee2010034, HQ600553. ***B. carinatus*** Hook. & Arn; California (USA), P.M. Peterson, CAN-19700 (2006), KM077294. ***B. catharticus*** Vahl (received as *B. araucanus* Phil.), PI 578719 RGI 441 (WRPIS) (Argentina), Keane 5 (ALTA), AY367954. ***B. catharticus*** Vahl., Collanzo, Asturias (Spain), A. Alonso and R. Noriega, LEB-132 (2011), KM077295. ***B. catharticus*** Vahl., Spain, Acedo, Acedo 92, KP987384. ***B. catharticus*** Vahl., Spain, Acedo, Acedo 23, KP987383. ***B. catharticus*** Vahl., MF063510. ***B. catharticus*** Vahl., MF508972. ***B. cebadilla*** Steud. (received as *B. coloratus*), PI 202696 (WRPIS) (Chile), Keane 13 (ALTA), AY367944. ***B. chrysopogon*** Viv., Iran, Acedo and Llamas, Acedo and Llamas 254, KP987320. ***B. ciliatus*** L., Quebec (Canada), Cayouette C8272 & Lavoie (DAO), AY367909. ***B. coloratus*** Steud., Region I (Chile), Peterson 15746 & Soreng (US), AY367943. ***B. commutatus*** Schrad., Spain, Acedo and Llamas, Llamas and Acedo 49, KP987322. ***B. commutatus*** Schrad., Spain, Acedo et al., Acedo et al. 219, KP987323. ***B. danthoniae*** Trin., PI 598455 TU85-028-0l (WRPIS) (Turkey), Keane 15 (ALTA), AY367941. ***B. danthoniae*** Trin., Iran, H. Mirzaie-Nodoushan, H. Mirzaie-Nodoushan 9, KP987329. ***B. danthoniae*** Trin., Iran, H. Mirzaie-Nodoushan, H. Mirzaie-Nodoushan 2, KP987328. ***B. diandrus*** Roth, L36509. ***B. diandrus*** Roth., CN 31600 PGR 2848 (PGRC) (Germany), Keane 25 (ALTA), AY367936. ***B. diandrus*** Roth., Brittany (France), EU036194 and EU036204. ***B. diandrus*** Roth, South Korea, YDK2009474, HQ600541. ***B. diandrus*** Roth, Beni Chiker, Nador (Morocco), J. Chrtek and Z. Docˇkalova, LEB - (2010), KM077297. ***B. diandrus*** Roth, Jeollanam-do, Suncheon-si (Korea), HCCN-PJ008548-PB-15, KF713200. ***B. diandrus*** Roth. var. *rigidus*, Brittany (France), EU036197 and EU036207. ***B. diandrus*** Roth, Iran, Alonso, Alonso 347, KP987437. ***B. diandrus*** Roth, Spain, Acedo and Llamas, Acedo and Llamas 91, KP987433. ***B. dolichocarpus*** Wagnon, Michoacán (Mexico), Peterson 16128 (US), AY367911. ***B. erectus*** Huds. (received as *B. benekenii* (Lange) Trimen), PI 337652 (WRPIS) (Turkey), Keane 8 (ALTA), AY367907. ***B. erectus*** Huds., France, FM956470. ***B. erectus*** Huds. Picos de Europa-Macizo Oriental, Cantabria (Spain), C. Acedo, A. Alonso, and F. Llamas, LEB-247(2011), KM077291. ***B. erectus*** Huds., Spain, Acedo, Acedo 208, KP987398. ***B. erectus*** Huds., Spain, Alonso and Noriega, Alonso and Noriega 188, KP987397. ***B. erectus*** Huds., Spain, Acedo et al., Acedo et al. 224, KP987399. ***B. erectus*** Huds., Ilhan Kaya, Muzaffer Mukemre, MW271027. ***B. erectus*** Huds., Ilhan Kaya, Muzaffer Mukemre, MW271028. ***B. erectus*** Huds., Ilhan Kaya, Muzaffer Mukemre, MW349611. ***B. exaltatus*** Bernh, Jalisco (Mexico), Peterson 16087 & Rosales (US), AY367912. ***B. fasciculatus*** C.Presl, Lahav (Israel), EU036193 and EU036203. ***B. firmior*** Stapf, South Africa, Panagos, Panagos 48, KP987426. ***B. frondosus*** (Shear) Woot. & Stand., Durango (Mexico), Peterson 15418 et al. (US), AY367913. ***B. grandis*** (Shear) Hitchc., California (USA), Cayouette 7947a (DAO), AY367914. ***B. gunckelli*** Matthei, Region I (Chile), Peterson 15697 & Soreng (US), AY367947. ***B. gunckelii*** Mathei, Chile, Peterson, Peterson 15613, KP987432. ***B. hordeaceus*** L. Calera de León, Badajoz (Spain), C. Acedo, F.M. Vazquez, A. Alonso and F. Llamas, LEB-109 (2011), KM077298. ***B. hordeaceus*** L., Greece, Raabe, Raabe, KP987343. ***B. hordeaceus*** L, Spain, Gutierrez and Vazquez, Gutierrez and Vazquez, KP987344. ***B. hordeaceus*** L., Germnay, Gregor, Gregor 3515, KP987334. ***B. incisus*** R.Otto & H.Scholz,, Germany, Otto, Otto, KP987345. ***B. inermis*** Leyss., Collected outside native range in Arizona (USA), Peterson 15295 & Cayouette (US), AY367915. ***Bromus inermis*** Leyss. Kyakhtinskii Raion, Buryatiya Republic (Russia), V. Chepinoga, LEB-28355 (2010),

KM077290. ***Bromus inermis*** Leyss, Russia, Chepinoga, Chepinoga, Bi2, KP987404. ***Bromus inermis*** Leyss, Russia, Chepinoga, Chepinoga, Bi1, KP987403. ***Bromus inermis*** Leyss, Spain, Alonso and Noriega, Alonso and Noriega 131, KP987409. ***Bromus inermis*** Leyss, Russia, Blinova, Blinova, KP987408. ***Bromus inermis*** Leyss, Ilhan Kaya, Muzaffer Mukemre, MW270937. ***B. intermedius*** Guss, Turkey, Donmez, Donmez 3350, KP987346. ***B. interruptus*** Duce, England, KP987347. ***B. japonicus*** Thunb. (received as *B. popovii* Drob.), PI 283198 CPl 24193 (WRPIS) (Russia), Keane 24 (ALTA), AY367940. ***B. japonicus*** Thunb., Hungary, Chrtek and Dockalova, Chrtek and Dockalova 18/1, KP987348. ***B. japonicus*** Thunb., Turkey, Aedo et al., Aedo et al. CA6570, KP987351. ***B. japonicus*** Thunb., Turkey, Donmez, Donmez 3371, KP987349. ***B. japonicus*** Thunb., Qinling Mountainsn (China), S.-L. Dong, MH711121. ***B. japonicus*** Thunb., China, X S Tang, TangXS0097, MH808795. ***B. kalmii*** A. Gray, CN 51222 C7099 (PGRC), Ontario (Canada), Keane 55 (ALTA), AY367916. ***B. kalmii*** A. Gray, Everwilde Farm (USA), C. Acedo, LEB-332 (2013), KM077292. ***B. kalmii*** A. Gray, Canada, M.J. Oldham and S.R. Brinker 37463, KP987410. ***B. kopetdagensis*** Drobow, Iran, H. Mirzaie-Nodoushan, H. Mirzaie-Nodoushan 7, KP987412. ***B. kopetdagensis*** Drobow, Iran, A. Alonso, A. Alonso 340, KP987411. ***. korotkoyi*** Drob., Inner Mongolia (China), Soreng 5160 et al. (US), AY367998. ***B. laevipes*** Shear, California (USA), Peterson 14840 et al. (US), AY367917. ***B. lanatipes*** (Shear) Rydb., Arizona (USA), Peterson 15270 & Cayouette (US), AY367918. ***B. lanatus*** Kunth, Region I (Chile), Peterson 15747 & Soreng (US), AY367919. ***B. lanceolatus*** Roth., Spain, Garcia and Vazquez, Garcia and Vazquez, KP987358. ***B. lanceolatus*** Roth, Spain, Acedo, Alonso and Llamas, Acedo, Alonso and Llamas 147, KP987356. ***B. lanceolatus*** Roth., Naderi, DU-000137, LC467156. ***B. lanceolatus*** Roth., Ribera de la Albuera, Badajoz (Spain), C. Acedo, F.M. Vazquez, A. Alonso and F. Llamas, LEB-101 (2011), KM077302. ***B. latiglumis*** (Shear) Hitchc., Ontario (Canada), Cayouette 4336-1 (DAO), AY367920. ***B. lithobius*** Trin., Chile, Carrasco-Falrias, Carrasco-Falrias 2, KP987385. ***B. madritensis*** L. subsp. ***rubens*** (L.) Husn., (1), Collected outside native range in Western Australia (Australia), Peterson 14534 et al. (US), AY367950. ***B. madritensis*** L. subsp. ***rubens*** (L.) Husn. (received as *B. madritensis*) (2), PI 239722 (WRPIS) (Iran), Keane 20 (ALTA), AY367938. ***B. madritensis*** L. subsp. ***rubens*** (L.) Husn. (received as *B. madritensis*), (3), PI 253735 (WRPIS) (Iraq), Keane 16 (ALTA), AY367937. ***B. madritensis*** L., EU036195 and EU036205. ***B. madritensis*** L., Spain, Acedo, Alonso and Llamas, Acedo, Alonso and Llamas 99, KP987435. ***B. madritensis*** L., Spain, Alonso, Alonso 348, KP987436. ***B. neglectus*** Nyman, Greece, Hageman, Scholz and Scharz, Scholz and Scharz, KP987359. ***B. marginatus*** Nees ex Steud., Oregon (USA), Soreng 6360 & Soreng (US), AY367921. ***B. modestus*** Renvoize, La Paz (Bolivia), Peterson 12639 et al. (US), AY367921. ***B. mucroglumis*** Wagnon, Arizona (USA), Peterson 15273 & Cayouette (US), AY367922. ***B. nervosus*** Acedo & Llamas, Spain, Garcia and Vazquez, Vazquez, KP987363. ***B. nervosus*** Acedo & Llamas, Portugal, Llamas, Acedo and Alonso, Llamas, Acedo and Alonso 9, KP987362. ***B. nottowayanus*** Fern., Illinois (USA), Chase 13512 (US), AY367923. ***B. nottowayanus*** Fern., U.S.A., Rothfels, Rothfels 2873, KP987413. ***B. pectinatus*** Thunb., PI 442453 (WRPIS) (Belgium), Keane 23 (ALTA), AY367939. ***B. pectinatus*** Thunb., South Africa, Aizpuru et al., Aizpuru et al. LM6093, KP987364. ***B. pellitus*** Rack., Santa Cruz (Argentina), Peterson 17267 et al. (US), AY367951. ***B. pflanzii*** Pilg., La Paz (Bolivia), Luteyn & Dorr 13828 (US), AY367924. ***B. porteri*** (Coult.) Nash, Arizona (USA), Peterson 15245 & Cayouette (US); AY367925. ***B. pseudodanthoniae*** Drobov, PI 204424 (WRPIS) (Turkey), Keane 21 (ALTA), AY367942. ***B. pseudolaevipes*** Wagnon, California (USA), Cayouette C7987 (DAO), AY367926. ***B. pseudolaevipes*** Wagnon, California (USA), EU036198 and EU036208. ***B. pseudodanthoniae*** Drobov,, Iran, Alonso, Alonso 341, KP987365. ***B. pubescens*** Muhl. ex Willd., Virginia (USA), Peterson 15776 & Saarela (US), AY367927. ***B. pubescens*** Muhl. ex Willd., U.S.A., Peterson, Peterson 15788, KP987414. ***B. pumilio*** (Trin.)P.M.Sm., Turkey, Metzger, Metzger, KP987313. ***B. pumilio*** (Trin.) P.M.Sm., Armenia, Nersesyan, Nersesyan 50-2004, KP987312. ***B. pumpellianus*** Scribn., PI 610833 (WRPIS) (Mongolia), Keane 17 (ALTA), AY367928. ***B. pumpellianus*** Scribn., Russia, Chepinoga, Chepinoga Bp1, KP987416. ***B. racemosus*** Schrad, Cofinal, León (Spain), A. Alonso, LEB- 29 (2011), KM077299. ***B. racemosus*** Schrad, Spain, Alonso, Alonso, Alonso 211, KP987367. ***B. racemosus*** Schrad, Spain, Acedo, Acedo 26, KP987366. ***B. ramosus*** Huds., Bulgaria, Aedo et al., Aedo et al., KP987418. ***B. ramosus*** Huds., UK, Keane 101 (ALTA), AY367929. ***B. ramosus*** Huds., Spain, Llamas et al. 79, KP987417. ***B. rechingeri*** Melderis, Iran, Alonso, Alonso 342, KP987368. ***B. richardsonii*** Link, Arizona (USA), Peterson 15282 & Cayouette (US), AY367930. ***B. riparius*** Rehmann, PI 598590 (WRPIS) (Czech Republic/Slovakia), Keane 26 (ALTA), AY367931. ***B. riparius***, Russia, Kiseleva, Kiseleva, KP987419. ***B. rubens*** L., Spain, Aedo et al., Acedo et al. 106, KP987440. ***B. rubens*** L., Morocco, Chrtek and Dockalova, Chrtek and Dockalova, KP987439. ***B. rubens*** L., EU036196 and EU036206. ***B. scoparius*** L., PI 204425 (WRPIS) (Turkey), Keane 28 (ALTA), AY367932. ***B. scoparius*** L., Turkey, Donmez, Donmez 1902, KP987374. ***B. scoparius*** L., Spain, Acedo, Alonso and Llamas, Alonso and Llamas 141, KP987373. ***B. secalinus*** L., Botanischer Garten Oldenburg (accession 07-104-07-74) (Germany), A. Alonso, LEB-339 (2011), KM077304. ***B. secalinus*** L., Germany, Alonso 345, KP987377. ***B. secalinus*** L., Russia, Shilova and Estarozhieva, Shilova and Estarozhieva, KP987376. ***B. secalinus*** L., Belgium, Andriessen and Vannerom Andriessen and Vannerom, KP987378. ***B. sitchensis*** Trin., U. S. A., Peterson, Peterson 19683, KP987386. ***B. speciosus*** Ness, South Africa, Catalan and Pimentel, Catalan and Pimentel SA043, KP996900. ***B.***

speciosus Ness, South Africa, Catalan and Pimentel SA043, KP996898. ***B. speciosus*** Ness, South Africa, Catalan and Pimentel, Catalan and Pimentel SA043, KP987429. ***B. speciosus*** Ness, South Africa, Catalan and Pimentel, Catalan and Pimentel SA043, KP987428. ***B. squarrosus*** L., Llombera, León (Spain), F. Llamas and C. Acedo, LEB-43 (2010), KM077303. ***B. squarrosus*** L., Spain, Llamas, Llamas 187, KP987381. ***B. squarrosus*** L., Hungary, Chrtek and Dockalova, Chrtek and Dockalova 1, KP987380. ***B. suksdorfii*** Vasey, U. S. A., Cronquist, Cronquist, KP987420. ***B. sterilis*** L., Philistean Plain, Sou (Israel), A. Danin, T. Raus, W. Sauer, S. Brullo, B. Valdes, F. Amich, S.G. Gardner, R.C.H.J. van Ham, A. Gambino, F. Axelrod, Battia Pazy and Rivka Nokrian (1981), SALA, KM077296. ***B. sterilis*** L., Brittany (France), EU036191 and EU036201. ***B. striatus*** Hitchc., PI 477988 974 (WRPIS) (France), Keane 6 (ALTA), AY367945. ***B. subvelutinus*** Shear., PI 392355 (WRPIS) (Uzbekistan), Keane 35 (ALTA), AY367953. ***B. suksdorfii*** Vasey, Washington (USA), Soreng 6352 & Soreng (US), AY367934. ***B. tectorum*** Huds., America, EU036192 and EU036202. ***B. tectorum*** Huds., Iran, Alonso, Alonso 343, KP987445. ***B. tectorum*** Huds., Czech Republic, Chrtek, Chrtek 3, KP987444. ***B. tectorum*** Huds., L36485. ***B. texensis*** (Shear) Hitchc., Texas (USA), Cayouette 668135 (DAO), AY367935. ***B. tomentellus*** Boiss. Turkey, Oezuedogru and Agar, Oezuedogru and Agar, KP987422. ***B. tomentellus*** Boiss., Turkey, Doenmez, Doenmez, 2178 and Guener, KP987423. ***Ampelodesmos mauritanicus*** (Poir.) T. Durand & Schinz, Menorca (Spain) Sa Mesquida, C. Acedo and F. Llamas; 253 (2011) LEB, KM077283. ***Anthoxanthum_ovatum*** Lag., Sardinia (Itaky) Iglesias M. Pimentel and E. Sahuquillo, (2009) SANT, KM077285. ***Danthoniastrum compactum*** (Boiss. & Heldr.) Holub, Mount Katarrachias, Thessaly (Greece), M. Röser, 10724 (2013) HAL, KM077282. ***Hordeum marinum*** Huds., Spain: Zamora, Villafafila, Zamora, Castilla y León, (Spain), C. Acedo and F. Llamas, 263 (2012) LEB, KM077287. ***Pleuropogon californicus*** (Nees) Vasey, California (U. S. A.) Solano D. Sanchez Mata and M.G. Barbour, (1996) MA, KM077284.

trnL (UAA):

Bromus anomalus Rupr. ex Fourn. (1) PI 232199 (WRPIS) (USA), Keane 49 (ALTA), AY367955. ***B. anomalus*** Rupr. ex Fourn. (2) Tamaulipas (Mexico), Peterson 15918 & Valdes-Reyna (US), AY367956. ***B. anomalus*** Rupr. ex Fourn., EU036183. ***B. anomalus*** Rupr. ex Fourn. Refulio-Rodriguez, Refulio-Rodriguez 145 (RSA), JF904751. ***B. arvensis*** L., EU036175. ***B. attenuatus*** Swallen, Tamaulipas and Nuevo León border (Mexico), Peterson 15926 & Valdes-Reyna (US), AY367960. ***B. berteroanus*** Colla, PI 224789 (WRPIS) (Chile), Keane 37 (ALTA), AY367994. ***B. brachyanthera*** Doll., La Paz (Bolivia), de Ros 9497 (US), AY367958. ***B. briziformis*** Fisch. & C.A.Mey., PI: 368 861, Former USSR, EU036182. ***B. carinatus*** Hook. & Arn., Kojima-shio-nasu, Kurashiki, Okayama Pref. (Japan), Kariyama KURA-8841 AB732921. ***B. carinatus*** Hook. & Arn., Durango (Mexico), Peterson 15421 et al. (US), AY367997. ***B. carinatus*** Hook. & Arn., Korea, J.H.Lee, J.H.Lee 2010034_TF, KX372390. ***B. catharticus*** Vahl, Tamashima-otoshima, Kurashiki, Okayama Pref. (Japan), T. Enomoto RIB-9448S RIB-30773, AB732922. ***B. catharticus*** Vahl (received as *B. araucanus* Phil.), PI 578719 RGI 441 (WRPIS) (Argentina), Keane 5 (ALTA), AY368003. ***B. catharticus*** Vahl., Canberra (Australia), EU036184. ***B. catharticus*** Vahl., Porto alegre (Brazil), ICN<BRA>:132.519, EU395890. ***B. catharticus*** Vahl, Giussani 354, DQ887428. ***B. catharticus*** Vahl, China, KP711142. ***B. catharticus*** Vahl, (Korea), KX372391. ***B. catharticus*** Vahl, Mason Mountain Wildlife Management Area, (U. S. A.), KL55, MG709440. ***B. cebadilla*** Steud. (received as *B. coloratus*), PI 202696 (WRPIS) (Chile), Keane 13 (ALTA), AY367993. ***B. ciliatus*** L., Quebec (Canada), Cayouette C8272 & Lavoie (DAO), AY367959. ***B. coloratus*** Steud., Region I (Chile), Peterson 15746 & Soreng (US), AY367992. ***B. commutatus*** Schrad., Kouyou-cho-higashi, Kobe, Hyogo Pref. (Japan), M. Mizuta KURA-47423, AB732923. ***B. danthoniae*** Trin., PI 598455 TU85-028-0l (WRPIS) (Turkey), Keane 15 (ALTA), AY367990. ***B. danthoniae*** Trin., PI: 254 874 (Afghanistan), EU036179. ***B. danthoniae*** Trin., Ghaen –Birjand, South Khorassan (Iran), Naderi 1210 (DU000145), LC467158. ***B. danthoniae*** Trin., Douleh, Mohsenabad Taheri, Khorassan Razavi (Iran), Naderi 1468(DU000138), LC467160. ***B. diandrus*** Roth., CN 31600 PGR 2848 (PGRC) (Germany), Keane 25 (ALTA), AY367985. ***B. diandrus*** Roth., Brittany (France), EU036172. ***B. diandrus*** Roth., Korea, YDK2009474_TF, KX372394. ***B. dolichocarpus*** Wagnon, Michoacán (Mexico), Peterson 16128 (US), AY367961. ***B. erectus*** Huds. (received as *B. benekenii* (Lange) Trimen), PI 337652 (WRPIS) (Turkey), Keane 8 (ALTA), AY367957. ***B. erectus*** Huds., EU119359. ***B. erectus*** Huds., Čertoryje meadow, Bílé Karpaty Mountains (Czech Republic), KU600345. ***B. erectus*** Huds., Čertoryje meadow, Bílé Karpaty Mountains (Czech Republic), KU600348. ***B. erectus*** Huds., Čertoryje meadow, Bílé Karpaty Mountains (Czech Republic), KU600387. ***B. erectus*** Huds., France, S. Coq, KY697471. ***B. erectus*** Huds., JX985261. ***B. exaltatus*** Bernh, Jalisco (Mexico), Peterson 16087 & Rosales (US), AY367962. ***B. frondosus*** (Shear) Woot. & Stand., Durango (Mexico), Peterson 15418 et al. (US), AY367963. ***B. grandis*** (Shear) Hitchc., California (USA), Cayouette 7947a (DAO), AY367964. ***B. gunckelli*** Matthei, Region I (Chile), Peterson 15697 & Soreng (US), AY367996. ***B. hordeaceus*** L., Okayama Harbor, Okayama Pref. (Japan), T. Enomoto RIB-15488S RIB-59663, AB732925. ***B. hordeaceaus*** L.,

EU036174. ***B. hordeaceus*** L., JQ041834. ***B. hordeaceus*** L., León, Castilla y León (Spain), Llamas, LEB146, KJ529407. ***B. hordeaceaus*** L., Białowieża Primeval Forest (Poland-Belarus), KX668092. ***B. hordeaceus*** L., France, S.Coq, KY697538. ***B. inermis*** Leyss., Collected outside native range in Arizona (USA), Peterson 15295 & Cayouette (US), AY367965. ***B. inermis*** Leyss., Canada, I. Hiiesalu, USAS:Hiiesalu 15, HM590248. ***B. inermis*** Leyss., Białowieża Primeval Forest (Poland-Belarus), KX668091. ***B. intermedius*** Guss, Algeria, Malika L. Ainouche, HQ130343. ***B. japonicus*** Thunb. (received as *B. popovii* Drob.), PI 283198 CPl 24193 (WRPIS) (Russia), Keane 24 (ALTA), AY367989. ***B. japonicus*** Thunb., Mason Mountain Wildlife Management Area, Texas (U. S. A.), KL58, MG709443. ***B. japonicus*** Thunb., Serbia, EU036181. ***B. japonicus*** Thunb., Korea, KX372392. ***B. japonicus*** Thunb., China, KP711141. ***B. kalmii*** A. Gray, CN 51222 C7099 (PGRC), Ontario (Canada), Keane 55 (ALTA), AY367966. ***B. kalmii*** A. Gray, EU119360. ***B. korotkoyi*** Drob., Inner Mongolia (China), Soreng 5160 et al. (US), AY367988. ***B. laevipes*** Shear, California (USA), Peterson 14840 et al. (US), AY367967. ***B. lanatipes*** (Shear) Rydb., Arizona (USA), Peterson 15270 & Cayouette (US), AY367968. ***B. lanatus*** Kunth, Region I (Chile), Peterson 15747 & Soreng (US), AY367969. ***B. lanceolatus*** Roth., Shush-Dezful, Khuzestan, (Iran) Naderi 1467 (DU000137), LC467161. ***B. latiglumis*** (Shear) Hitchc., Ontario (Canada), Cayouette 4336-1 (DAO), AY367970. ***B. madritensis*** L., EU036170. ***B. madritensis*** L. subsp. ***rubens*** (L.) Husn., (1), Collected outside native range in Western Australia (Australia), Peterson 14534 et al. (US), AY367987. ***B. madritensis*** L. subsp. ***rubens*** (L.) Husn. (received as *B. madritensis*) (2), PI 239722 (WRPIS) (Iran), Keane 20 (ALTA), AY367986. ***B. madritensis*** L. subsp. ***rubens*** (L.) Husn. (received as *B. madritensis*), (3), PI 253735 (WRPIS) (Iraq), Keane 16 (ALTA), AY367986. ***B. marginatus*** Nees ex Steud., Oregon (USA), Soreng 6360 & Soreng (US), AY367971. ***B. mucroglumis*** Wagnon, Arizona (USA), Peterson 15273 & Cayouette (US), AY367972. ***B. pectinatus*** Thunb., PI 442453 (WRPIS) (Belgium), Keane 23 (ALTA), AY367988. ***B. pellitus*** Rack., Santa Cruz (Argentina), Peterson 17267 et al. (US), AY368000. ***B. pflanzii*** Pilg., La Paz (Bolivia), Luteyn & Dorr 13828 (US), AY367973. ***B. porteri*** (Coult.) Nash, Arizona (USA), Peterson 15245 & Cayouette (US), AY367974. ***B. pseudodanthoniae*** Drobov, PI 204424 (WRPIS) (Turkey), Keane 21 (ALTA), AY367991. ***B. pseudodanthoniae*** Drobov, 14PI: 219 999 (Afghanistan), EU036180. ***B. pseudolaevipes*** Wagnon, California (USA), Cayouette C7987 (DAO), AY367975. ***B. pubescens*** Muhl. ex Willd., Virginia (USA), Peterson 15776 & Saarela (US), AY367976. ***B. pumilio*** (Trin.)P.M.Sm., Shirvan -Quchan, North Khorassan (Iran), Naderi1336 (DU000139), LC467162. ***B. pumpellianus*** Scribn., PI 610833 (WRPIS) (Mongolia), Keane 17 (ALTA), AY367977. ***B. ramosus*** Huds., UK, Keane 101 (ALTA), AY367978. ***B. ramosus*** Huds., Hodkinson41 TCD, EF137595. ***B. richardsonii*** Link, Arizona (USA), Peterson 15282 & Cayouette (US), AY367979. ***B. riparius*** Rehmann, PI 598590 (WRPIS) (Czech Republic/Slovakia), Keane 26 (ALTA), AY367980. ***B. rubens*** L., Bibans (Algeria), EU036169. ***B. rubens*** L., MG657033. ***B. rubens*** L., MG657046. ***B. scoparius*** L., PI 204425 (WRPIS) (Turkey), Keane 28 (ALTA), AY367981. ***B. scoparius*** L., EU036176. ***B. secalinus*** L. Okayama Harbor, Okayama Pref. (Japan), H. Kobatake KURA-8415, AB732927. B. ***squarrosus*** L., EU036173. B. ***squarrosus*** L., Castilla y León (Spain), Llamas, LEB WP13, KJ529263. ***B. striatus*** Hitchc., PI 477988 974 (WRPIS) (France), Keane 6 (ALTA), AY367994. ***B. subvelutinus*** Shear., PI 392355 (WRPIS) (Uzbekistan), Keane 35 (ALTA), AY368002. ***B. suksdorfii*** Vasey, Washington (USA), Soreng 6352 & Soreng (US), AY367983. ***B. suksdorfii*** Vasey, EU036187. ***B. tectorum*** Huds., Korea, KX372395. ***B. texensis*** (Shear) Hitchc., Texas (USA), Cayouette 668135 (DAO), AY367984. ***Ampelodesmos mauritanicus*** (Poir.)T. Durand & Schinz, Cultivated in Utah from material of the Mediterranean (U. S. A.), M.E. Barkworth 2007.001 UTC 246449, EU200838. ***Anthoxanthum ovatum*** Lag., Cáceres, Extremadura (Spain), UZ 24.07, KX580278. ***Danthoniastrum compactum*** (Boiss. & Heldr.) Holub, Soreng, Soreng7520-1 (US), GU254958, ***Hordeum marinum*** Huds., USDA PI 401364, KF600707.

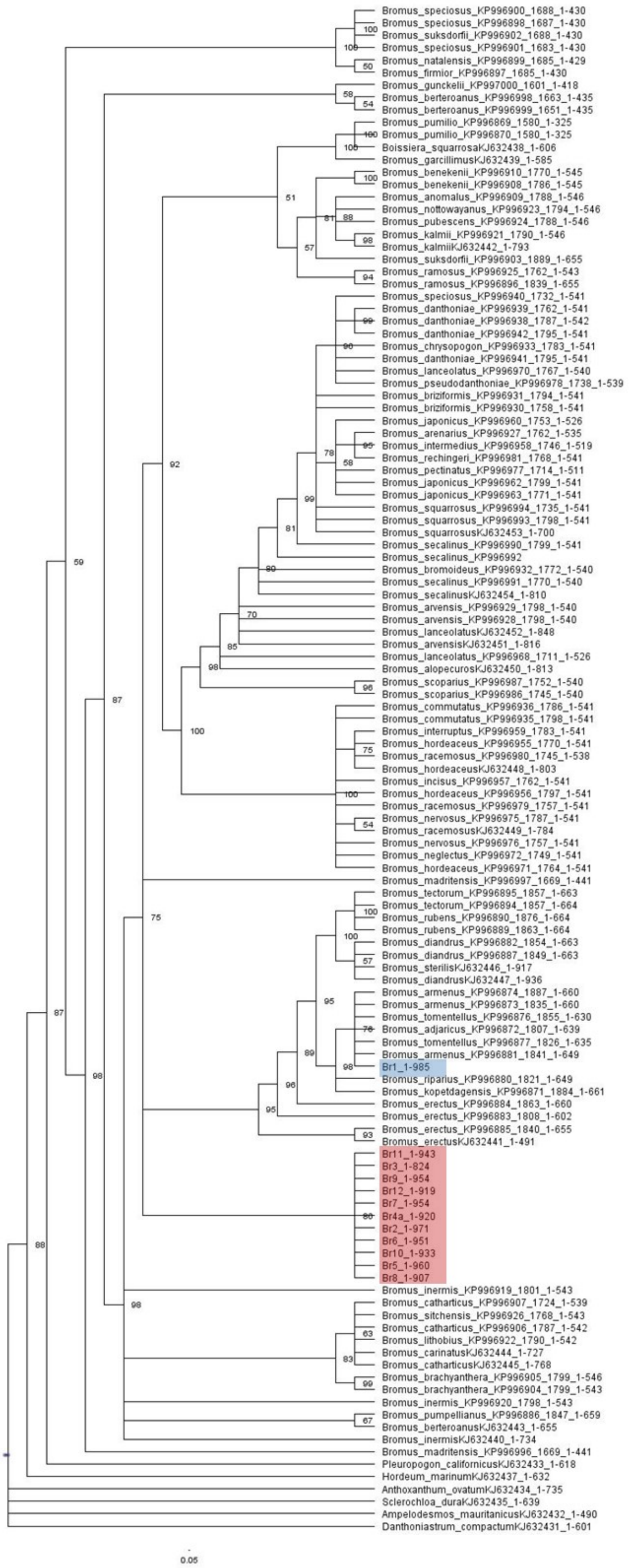

Figure S1. Consensus phylogenetic tree obtained from the BI analyses based on the nuclear marker ETS without the large indel. The numbers over the branches represents the bootstrap values from the ML analysis and the number under the branches correspond to the posterior probabilities (PP) form the BI analysis. Br: new *Bromus* samples generated in this study. Br samples from Picos de Europa and the Cantabrian Mountains have been highlighted in red. The Br sample from lower lands has been highlighted in blue.

References

Alonso A, Bull RD, Acedo C, Gillespie LJ (2014) Design of plant-specific PCR primers for the ETS region with enhanced specificity for tribe Bromeae and their application to other grasses (Poaceae). Botany 92:693–699. https://doi.org/10.1139/cjb-2014-0062.

Barkworth ME, Arriaga MO, Smith JF, Jacobs SW, Valdés-Reyna J, Bushman BS (2008) Molecules and morphology in South American stipeae (Poaceae). Syst Bot 33:719–731. https://doi.org/10.1600/036364408786500235.

Bouchenak-Khelladi Y, Verboom AG, Hodkinson TR, Salamin N, Francois O, Chonghaile NG, Savolainen V (2009) The origins and diversification of C4 grasses and savanna-adapted ungulates. Glob Chang Biol 15:2397–2417. https://doi.org/10.1111/J.1365-2486.2009.01860.X.

Chumová Z, Záveská E, Mandáková T, Krak K, Trávníček P (2017) The Mediterranean: the cradle of *Anthoxanthum* (Poaceae) diploid diversity. Ann Bot 120:285–302. https://doi.org/10.1093/AOB/MCX021.

Cialdella AM, Giussani LM, Aagesen L, Zuloaga FO, Morrone O (2007) A phylogeny of *Piptochaetium* (Poaceae: Pooideae: Stipeae) and related genera based on a combined analysis including trnL-F, rpl16, and morphology. Syst Bot 32:545–559. https://doi.org/10.1600/036364407782250607.

Essi L, Longhi-Wagner HM, de Souza-Chies TT (2008) Phylogenetic analysis of the *Briza* Complex (Poaceae). Mol Phylogenet Evol 47:1018–1029. https://doi.org/10.1016/J.YMPEV.2008.03.007.

Fortune PM, et al., (2008) Molecular phylogeny and reticulate origins of the polyploid *Bromus* species from section Genea (Poaceae). Am J Bot 95:454–464. https://doi.org/10.3732/ajb.95.4.454.

Hiiesalu I, Oepik M, Metsis M, Lilje L, Davison J, Vasar M., Moora M, Zobel M, Wilson SD, Paertel M (2012) Plant species richness belowground: higher richness and new patterns revealed by next-generation sequencing. Mol Ecol 21:2004–2016. https://doi.org/10.1111/J.1365-294X.2011.05390.X.

Holá E, Kocková J, Těšitel J (2017) DNA barcoding as a tool for identification of host association of root-hemiparasitic plants. Folia Geobot 52:227–238. https://doi.org/10.1007/S12224-017-9286-Z/TABLES/2.

Hsiao C, Chatterton NJ, Asay KH, Jensen KB (1995) Phylogenetic relationships of the monogenomic species of the wheat tribe, Triticeae (Poaceae), inferred from nuclear rDNA (internal transcribed spacer) sequences. Genome 38:211–223. https://doi.org/10.1139/G95-026.

Kowalczyk R, Wójcik JM, Taberlet P, Kamiński T, Miquel C, Valentini A, Craine JM, Coissac E (2019) Foraging plasticity allows a large herbivore to persist in a sheltering forest habitat: DNA metabarcoding diet analysis of the European bison. For Ecol Manage 449:117474. https://doi.org/10.1016/J.FORECO.2019.117474.

Mason-Gamer RJ (2013) Phylogeny of a genomically diverse group of *Elymus* (Poaceae) allopolyploids reveals multiple levels of reticulation. PLoS One 8:e78449. https://doi.org/10.1371/JOURNAL.PONE.0078449.

Matsushima R, Yamashita J, Kariyama S, Enomoto T, Sakamoto W (2013) A Phylogenetic re-evaluation of morphological variations of starch grains among Poaceae species. J Appl Glycosci 60:37–44. https://doi.org/10.5458/JAG.JAG.JAG-2012_006.

Pimentel M, Escudero M, Sahuquillo E, Minaya MÁ, Catalán P (2017) Are diversification rates and chromosome evolution in the temperate grasses (Pooideae) associated with major environmental changes in the Oligocene-Miocene? PeerJ 2017:e3815. https://doi.org/10.7717/PEERJ.3815/SUPP-8.

Pourmoshir Z, Amirahmadi A, Naderi R (2019) An overview od the phylogenetic relationships of *Bromus pumilio* (Poaceae) and allies based on nrDNA ITS and trnL-F sequences. Iran J Bot 25:2019. https://doi.org/10.22092/ijb.2019.124046.1221.

Refulio-Rodriguez NF, Columbus JT, Gillespie LJ, Peterson PM, Soreng RJ (2012) Molecular phylogeny of *Dissanthelium* (Poaceae: Poçoideae) and its taxonomic implications. Syst Bot 37:122–133. https://doi.org/10.1600/036364412X616701.

Romaschenko K, Peterson PM, Soreng RJ, Garcia-Jacas N, Susanna A (2010) Phylogenetics of Stipeae 511 phylogenetics of Stipeae (Poaceae: Pooideae) based on plastid and nuclear DNA sequences. In: In: Seberg O,

Petersen G, Barfod AS, Davis JI eds. Diversity, phylogeny, and evolution in the monocotyledons. Aarhus: Aarhus University Press pp 511–538.

Saarela JM, Peterson PM, Keane RM, Cayouette J, Graham SW (2007) Molecular phylogenetics of *Bromus* (Poaceae: Pooideae) based on chloroplast and nuclear DNA sequence data. Aliso 23: 450–467.

Schardl CL, Craven KD, Speakman S, Stromberg A, Lindstrom A, Yoshida R (2008) A novel test for host-symbiont codivergence indicates ancient origin of fungal endophytes in grasses. Syst Biol 57:483–498. https://doi.org/10.1080/10635150802172184.

Wallinger C, Juen A, Staudacher K, Schallhart N, Mitterrutzner E, Steiner EM, Thalinger B, Traugott M (2012) Rapid Plant identification using species- and group-specific primers targeting chloroplast DNA. PLoS One 7:e29473. https://doi.org/10.1371/JOURNAL.PONE.0029473.

Yao PC, Gao HY, Wei YN, Zhang JH, Chen XY, Li HQ (2017) Evaluating sampling strategy for DNA barcoding study of coastal and inland halo-tolerant Poaceae and Chenopodiaceae: A case study for increased sample size. PLoS One 12:e0185311. https://doi.org/10.1371/JOURNAL.PONE.0185311.
